# Natural Antioxidant-Derived Compounds and Materials as Emerging MALDI Matrices: Applications, Evidence, and Perspectives

**DOI:** 10.3390/ijms27209285

**Published:** 2026-10-19

**Authors:** Katarzyna Bania, Dorota Bartusik-Aebisher, Klaudia Dynarowicz, Barbara Smolak, David Aebisher

**Affiliations:** 1 Medical Science Club, Faculty of Medicine, University of Rzeszów, 35-310 Rzeszów, Poland; kb135595@stud.ur.edu.pl; 2 Department of Biochemistry and General Chemistry, Faculty of Medicine, University of Rzeszów, 35-310 Rzeszów, Poland; dbartusikaebisher@ur.edu.pl (D.B.-A.); kdynarowicz@ur.edu.pl (K.D.); 3 Department of Diagnostic Imaging and Nuclear Medicine, Faculty of Medicine, University of Rzeszów, 35-310 Rzeszów, Poland; bsmolak@ur.edu.pl; 4 Department of Photomedicine and Physical Chemistry, Faculty of Medicine, University of Rzeszów, 35-310 Rzeszów, Poland

**Keywords:** antioxidants, MALDI-MS, MALDI-TOF-MS, natural products, phenolic compounds, flavonoids, low-molecular-weight compounds, MALDI matrices, alternative matrices, mass spectrometry imaging

## Abstract

Antioxidants constitute a chemically diverse group of compounds that are widely studied in food, natural products, and biological systems, creating a continuing need for analytical methods capable of their reliable characterization. Matrix-assisted laser desorption/ionization mass spectrometry (MALDI-MS) provides rapid molecular analysis with minimal sample preparation and has been increasingly applied to low-molecular-weight compounds, natural products, food constituents, and biological samples. However, the analysis of small molecules by MALDI-MS is often limited by matrix-derived background signals, differences in ionization efficiency, heterogeneous crystallization, and difficulties in achieving reliable quantitative measurements, highlighting the need for alternative matrix systems with improved and more controllable analytical properties. In this context, naturally occurring antioxidants are of particular interest because many possess aromatic and conjugated structures, UV-absorbing groups, acid–base and redox properties, and functional groups capable of matrix–analyte interactions, which may support their function as MALDI matrices. This review summarizes the use of MALDI-MS for the analysis of antioxidant compounds in purified samples, plant extracts, food products, and biological materials, while critically evaluating the emerging concept of using naturally occurring antioxidants as alternative MALDI matrices. Particular emphasis is placed on the structural and physicochemical features relevant to matrix performance, including aromaticity, UV absorption, acid–base properties, redox behavior, crystallization, and matrix–analyte interactions. Conventional organic matrices are discussed alongside emerging alternatives, including ionic liquid matrices, nanomaterial-based systems, carbon-based materials, and matrix-free approaches. Importantly, the available evidence indicates that only a limited number of natural antioxidant-derived compounds and materials have been experimentally demonstrated as MALDI matrices, whereas evidence for other candidates remains preliminary or theoretical. Their potential analytical value is further considered in relation to reproducibility, background interference, matrix selectivity, and quantitative performance, together with future opportunities involving rational matrix design, mass spectrometry imaging, and computational approaches. Natural antioxidant-derived compounds and materials therefore represent an emerging and still insufficiently explored class of MALDI matrices that could expand the range of matrix chemistries available for small-molecule analysis, although their analytical value will ultimately depend on compound-specific experimental validation rather than antioxidant activity alone.

## 1. Introduction

Oxidative stress contributes to numerous chronic diseases and has an effect on food quality, nutritional value, and shelf life, making antioxidants important analytical targets in biomedical, pharmaceutical and food research [1,2]. Naturally occurring antioxidants are structurally diverse, and include phenolic acids, flavonoids, tannins, carotenoids, and vitamins, making their identification and quantification in complex biological and natural matrices challenging [1,3]. High-performance liquid chromatography (HPLC) and liquid chromatography–mass spectrometry (LC-MS) remain widely used for antioxidant profiling because of their sensitivity and compatibility with chemically diverse analytes, while gas chromatography–mass spectrometry (GC-MS) and nuclear magnetic resonance (NMR) provide complementary analytical information [2,3].

Among modern mass spectrometric techniques, matrix-assisted laser desorption/ionization mass spectrometry (MALDI-MS) has developed into a versatile analytical platform capable of rapid molecular characterization with minimal sample preparation [4]. Since its introduction, MALDI-MS has become an important tool for the analysis of proteins and other biomacromolecules and has subsequently been applied in numerous fields, including metabolomics, lipidomics, food chemistry, natural product research, pharmaceutical analysis, and mass spectrometry imaging [4,5]. Unlike many separation-based approaches, MALDI-MS offers a high analytical throughput while enabling the analysis of thermally labile and fragile molecules through soft ionization, making it attractive for the characterization of complex biological and plant-derived samples [4,5,6].

The analytical performance of MALDI-MS, however, strongly depends on the properties of the selected matrix. Conventional organic matrices, including α-cyano-4-hydroxycinnamic acid (CHCA), 2,5-dihydroxybenzoic acid (DHB), and sinapinic acid (SA), provide effective ionization for numerous applications but are also associated with several well-recognized limitations [4,5]. Matrix-derived background ions can interfere with the detection of low-molecular-weight analytes, particularly below *m*/*z* 500, while heterogeneous crystallization and variable co-crystallization between the matrix and analyte may reduce reproducibility and contribute to the so-called “sweet spot” phenomenon [5]. These limitations continue to stimulate the search for alternative matrix materials that may provide improved ionization efficiency, reduced background interference, enhanced reproducibility, and better compatibility with small-molecule analysis [4,5,6].

Over the past several years, increasing attention has been directed toward naturally occurring compounds as having potential. However, a natural origin itself is not enough to establish that a matrix is environmentally friendly or sustainable. The evaluation of the environmental profile of an analytical procedure should be supported by the basis of the complete workflow, including factors such as solvent use, energy consumption, waste generation, toxicity, and sample-preparation requirements, rather than inferred from the biological origin of the matrix. Therefore, we should not inherently consider natural antioxidant-derived matrices “green” unless their environmental performance has been systematically assessed using appropriate green analytical chemistry principles or metrics [7,8]. Importantly, the concept of antioxidant-derived MALDI matrices must be distinguished from previously known instances where antioxidant compounds serve as MALDI matrices. A range of phenolic acids—including caffeic, ferulic, and sinapinic acids—exhibit structural features typical of effective MALDI matrices and have been the subject of research or practical application in mass spectrometry analysis. Their aromatic systems and conjugated bonds facilitate ultraviolet radiation absorption, while their functional groups (phenolic and carboxylic) can participate in matrix-analyte interactions and ionization processes. Sinapinic acid, in particular, is an established MALDI matrix, demonstrating that antioxidant activity and matrix functionality can coexist within a single molecule. Consequently, the innovative nature of research into natural antioxidants for MALDI-MS should not be defined solely by their antioxidant properties. Rather, the essence of this research lies in the systematic evaluation of compounds not previously used as MALDI matrices and in determining how their structural and physicochemical properties influence ionization efficiency, background signal generation, crystallization, and analyte selectivity. This distinction is crucial for the correct interpretation of available data and prevents the erroneous classification of established MALDI matrices as novel, antioxidant-derived alternatives. Natural antioxidants are particularly interesting candidates because many possess physicochemical characteristics relevant to MALDI ionization, including aromatic or conjugated systems, ultraviolet absorption, and proton-donating functional groups that may contribute to laser energy absorption and ion formation [5,6]. At the same time, these compounds remain important analytical targets in studies of food quality, plant metabolomics, nutritional sciences, and human health [1,2]. Thus, the role of MALDI-MS in antioxidant research encompasses both the characterization of antioxidant molecules and the investigation of naturally derived compounds as potential functional components of the analytical process [4,5,6].

**Figure 1:**
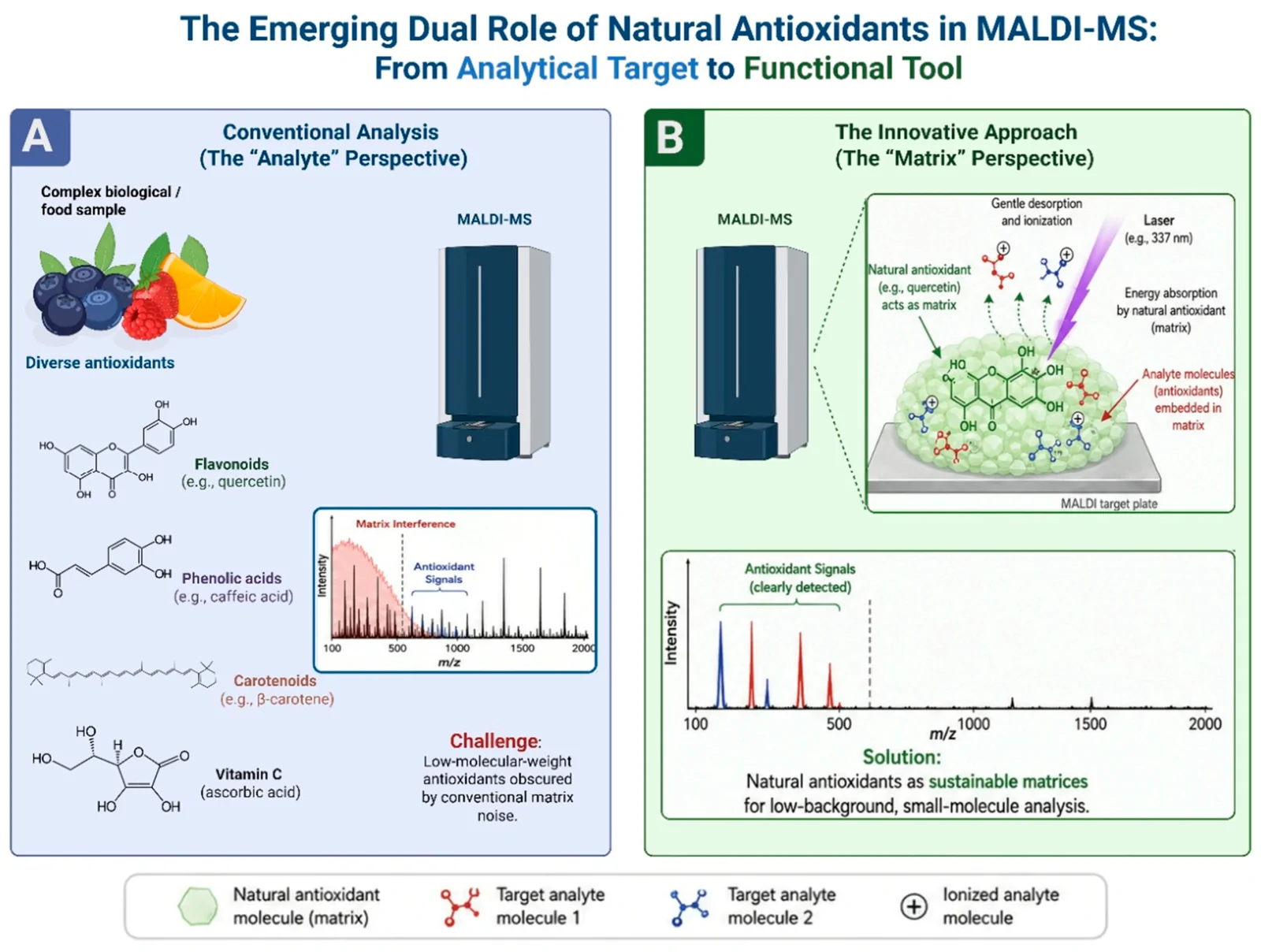
The emerging dual role of natural antioxidants in MALDI-MS: from analytical target to functional tool. (**A**) Conventional ‘analyte’ perspective: antioxidants are the targets of analysis but may be affected by matrix-derived background interference. (**B**) Alternative ‘matrix’ perspective: selected natural antioxidants are investigated as potential MALDI matrices capable of absorbing laser energy and facilitating analyte ionization while potentially reducing background interference. Created in BioRender. Bania, K. (2026) https://BioRender.com/vzxpu8k.

The dual role of natural antioxidants in MALDI-MS—as both analytical targets and potential matrix materials—is conceptually illustrated in Figure 1. In the conventional analytical workflow (Panel A), antioxidants present in complex biological and food matrices are the targets of analysis; however, their detection may be affected by matrix-derived background interference from conventional organic matrices, particularly in the low-*m*/*z* region. In the alternative approach (Panel B), selected natural antioxidants are investigated as potential MALDI matrices on the basis of physicochemical properties such as aromatic conjugation and UV absorption, which may support laser energy absorption, analyte desorption, and ionization while potentially reducing background interference. This dual functionality, in which compounds from the same broad chemical group can be investigated both as analytes and as components of the MALDI ionization system, represents an emerging analytical concept that provides the basis for evaluating natural antioxidants as alternative MALDI matrices.

The aim of this review is to provide a comprehensive overview of the application of MALDI-MS for the analysis of antioxidants and natural products, with particular emphasis on the development and evaluation of natural antioxidant-derived matrices for low-molecular-weight analysis. Particular focus is given to experimentally demonstrated antioxidant-derived matrices, their comparison with conventional MALDI matrices, relevant mechanistic considerations, analytical limitations, and quantitative validation. Applications in food and natural-product analysis are also considered due to their direct relevance to antioxidant-rich samples.

## 2. Fundamentals of MALDI-MS

### 2.1. Principle of MALDI Ionization

Matrix-assisted laser desorption/ionization (MALDI) is a soft ionization technique widely used for the analysis of thermally labile and high-molecular-weight compounds with minimal fragmentation. Since its introduction by Karas and Hillenkamp in 1988 [9], MALDI has expanded from protein analysis to applications in proteomics, metabolomics, lipidomics, pharmaceutical sciences, food chemistry, and mass spectrometry imaging [10,11]. Despite decades of research, the mechanisms of MALDI ion formation remain only partly understood because desorption and ionization involve multiple processes occurring on nanosecond timescales [5,6]. Current evidence indicates that ion formation results from interconnected photophysical and photochemical events, including laser-induced matrix excitation, energy redistribution, plume expansion, and gas-phase ion–molecule reactions [5,12].

The matrix plays a central role in these processes. It absorbs laser radiation, facilitates energy transfer to the analyte, and helps limit thermal degradation during desorption [4,13]. Proton transfer, cation attachment, electron transfer, and transient clustering may all contribute to ion formation, depending on the physicochemical properties of the matrix and analyte [12,14]. Consequently, matrix selection strongly influences ionization efficiency, signal intensity, fragmentation behavior, and analytical reproducibility [4,13,15].

Matrix performance is also related to optical properties and laser wavelength. Conventional organic matrices typically absorb strongly in the ultraviolet region and are compatible with nitrogen lasers at 337 nm or frequency-tripled Nd:YAG lasers (ALPHALAS GmbH, Göttingen, Germany) at 355 nm [4,13,16,17]. The efficient overlap between matrix absorption and laser wavelength promotes energy deposition and analyte desorption [6,13]. However, analytical performance is also affected by the crystal morphology, matrix-to-analyte ratio, laser fluence, sample homogeneity, and co-crystallization efficiency [6,15,16]. These factors contribute to the variability between MALDI preparations and remain important considerations in matrix development.

MALDI-MS can operate in positive or negative ion mode, with the preferred polarity depending on the analyte chemistry [11,14]. Positive ion mode commonly produces ions such as [M+H]^+^, [M+Na]^+^, and [M+K]^+^ [4,8], whereas negative ion mode is particularly useful for acidic compounds, including phenolic acids, phospholipids, sulfated metabolites, nucleotides, and several classes of natural products [14,17]. Advances in matrix chemistry and ionization strategies have expanded the use of negative-ion MALDI-MS for low-molecular-weight metabolites that may show limited sensitivity or substantial matrix interference in positive mode [15,17,18]. Ionization polarity should therefore be considered together with matrix selection and laser conditions because these parameters directly affect sensitivity, selectivity, and spectral quality [13,15,18].

**Figure 2:**
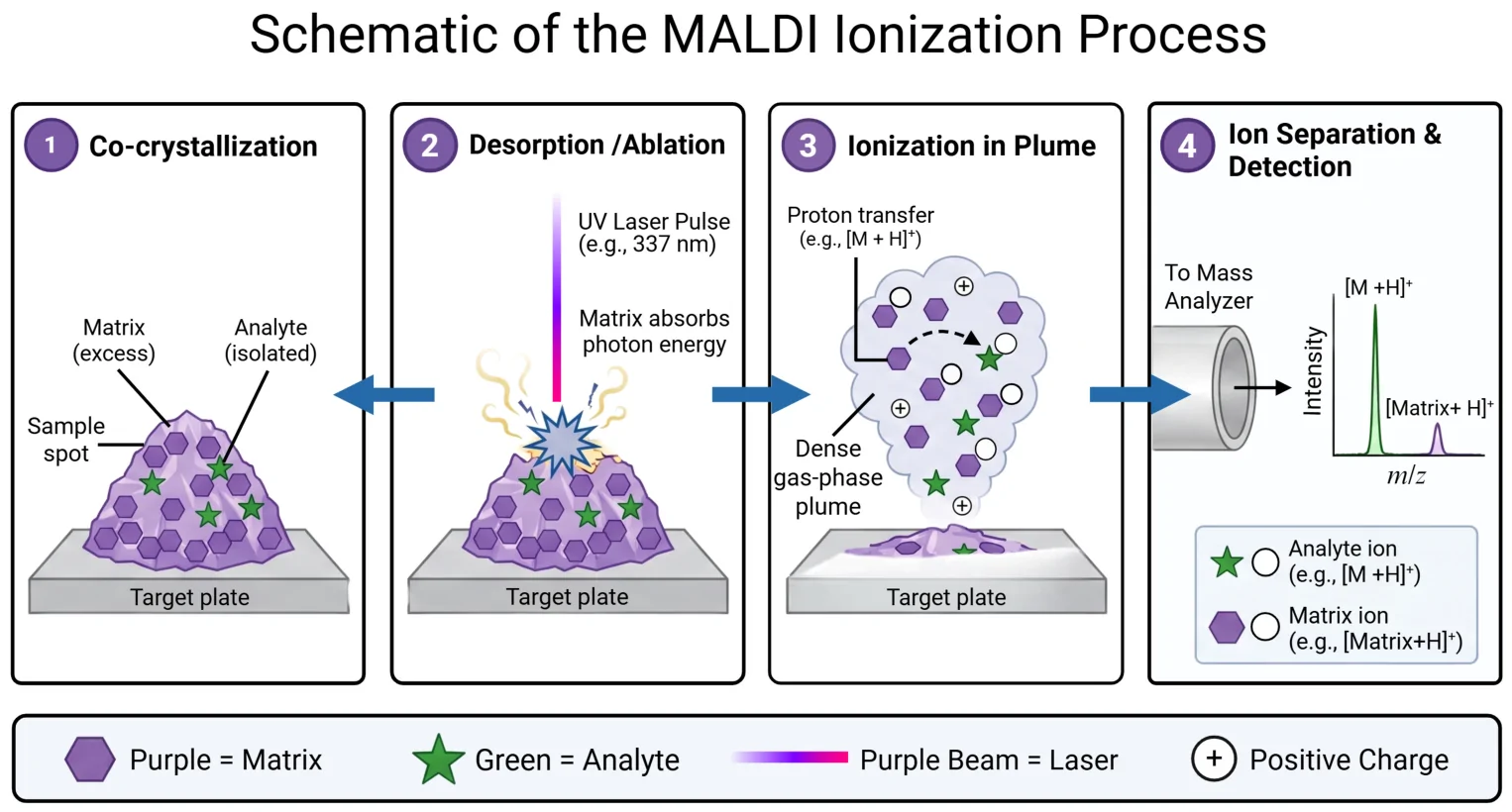
Schematic representation of the MALDI ionization process. The main stages include matrix–analyte co-crystallization, laser-induced desorption and ionization, gas-phase ion formation, and ion separation and detection. Created in BioRender. Bania, K. (2026) https://BioRender.com/s2r6vgc.

The main stages of the MALDI process are illustrated in Figure 2, including matrix–analyte co-crystallization, laser-induced desorption and ionization, and subsequent ion separation and detection. Matrix selection is particularly important for low-molecular-weight analysis because matrix-derived ions may interfere with analyte signals in the low-*m*/*z* region.

### 2.2. Conventional MALDI Matrices

The analytical performance of MALDI-MS is strongly dependent on matrix selection. An ideal MALDI matrix should efficiently absorb laser radiation, promote homogeneous co-crystallization with the analyte, facilitate ion formation, generate minimal chemical background, and remain stable under vacuum conditions [4,10]. Although many compounds have been investigated as potential matrices, only a relatively small number of organic matrices are widely used because of their reliable performance across different analyte classes [4,5]. Among the most established are α-cyano-4-hydroxycinnamic acid (CHCA), 2,5-dihydroxybenzoic acid (DHB), and sinapinic acid (SA), which also serve as reference matrices for the evaluation of new matrix systems [4,12]. Their main properties, applications, and limitations are summarized in Table 1.

CHCA is commonly used for peptides and other moderately low-molecular-weight compounds because of its high ionization efficiency and ability to form relatively small, homogeneous crystals [5,15]. These properties often support high sensitivity and good spectral resolution. However, CHCA can generate abundant matrix-derived ions in the low-*m*/*z* region, which may interfere with the detection of small metabolites and other low-molecular-weight analytes [4,19,20]. DHB has broader applicability and is widely used for peptides, lipids, glycans, oligosaccharides, and natural products. Its relatively gentle ionization behavior is associated with reduced fragmentation and the improved detection of labile molecules [15,21]. However, DHB often forms large, heterogeneous crystals, which may lead to uneven analyte distribution, reduced spatial resolution in imaging experiments, and increased spot-to-spot variability known as the “sweet spot” effect [4,10,21].

Sinapinic acid is primarily used for intact proteins and other high-molecular-weight analytes. Compared with CHCA, SA generally favors the formation of intact molecular ions with reduced fragmentation, making it suitable for protein profiling and top-down applications [4,12,15]. Despite their different preferred applications, CHCA, DHB, and SA share several physicochemical characteristics, including efficient UV absorption, aromatic conjugation, and acidic functional groups capable of participating in proton-transfer reactions during ion formation [4,9]. These properties contribute to their continued use in routine MALDI analysis.

Conventional organic matrices nevertheless present important limitations in low-molecular-weight analysis. Matrix-derived ions can overlap with analyte signals below *m*/*z* 500, reducing spectral clarity and limiting sensitivity for metabolites, pharmaceuticals, antioxidants, and other small molecules [4,6,19]. In addition, the heterogeneous crystallization, variable crystal morphology, and inconsistent matrix–analyte co-crystallization can reduce shot-to-shot reproducibility and quantitative reliability [10,20,22]. These effects are particularly relevant in MALDI imaging, where the crystal size influences spatial resolution and molecular localization [19,22]. Consequently, several approaches have been developed to improve matrix performance, including binary matrices, ionic liquid matrices, matrix additives, and chemically modified derivatives [4,5,21].

Despite these developments, background interference and reproducibility remain important limitations of conventional matrix systems. This has encouraged the investigation of alternative materials with different optical, chemical, and ionization properties for low-molecular-weight analysis [4,6,21]. Naturally occurring compounds are among the candidates being explored because of their structural diversity, photochemical properties, and potential relevance to sustainability-oriented analytical approaches. This has also stimulated interest in natural antioxidants as possible functional MALDI matrices, although their performance and environmental profile require compound-specific experimental validation and assessment of the associated preparation workflow [4,7,8].

**Figure 3:**
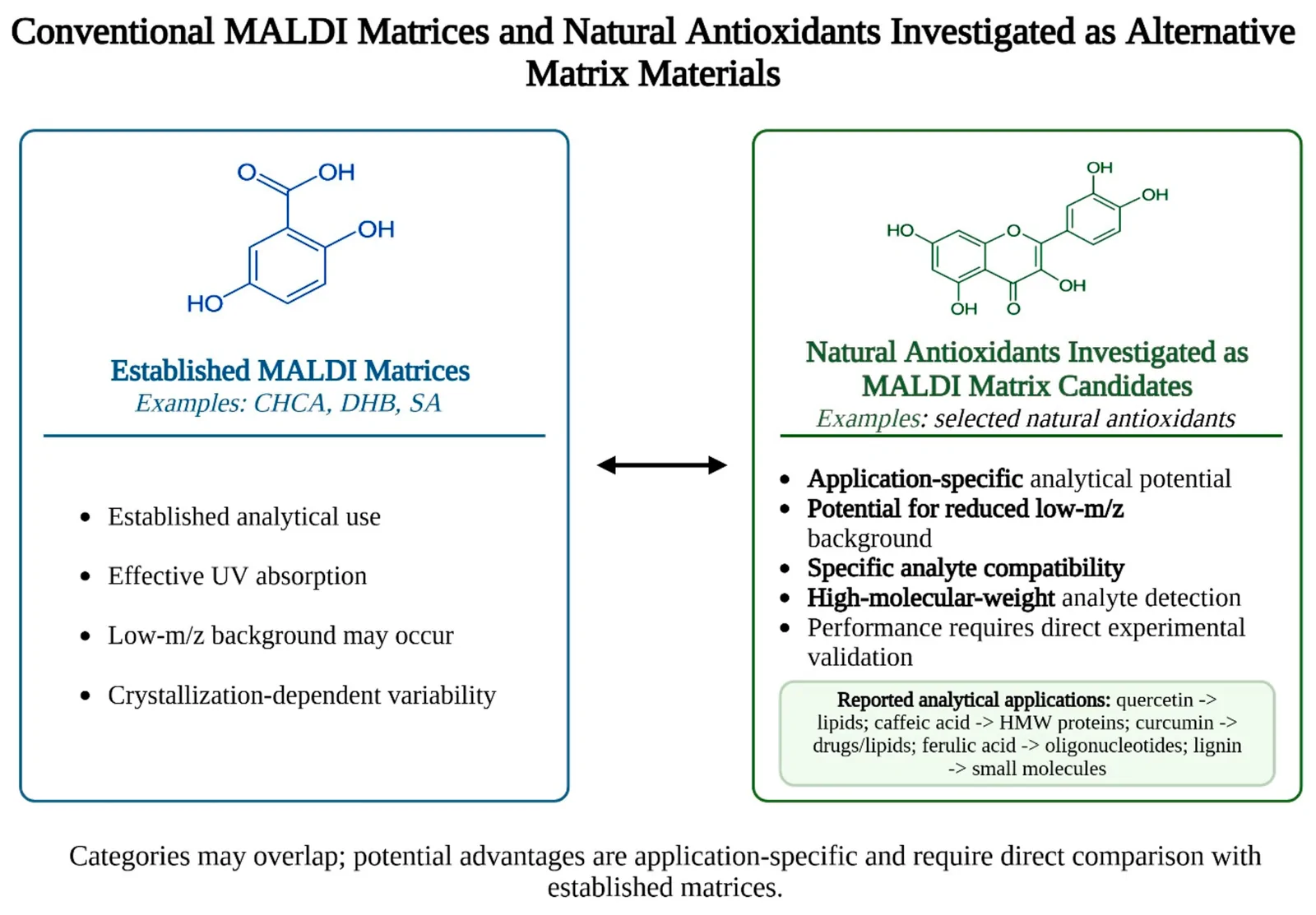
Conventional MALDI matrices and natural antioxidants investigated as alternative matrix materials. Conventional MALDI matrices and antioxidant-derived matrix candidates are not mutually exclusive categories, as established MALDI matrices such as DHB may also exhibit antioxidant properties. The distinction reflects their established analytical use versus their investigation as alternative matrix materials derived from natural antioxidants. Potential analytical advantages remain analyte- and method-dependent and require direct experimental validation. Created in BioRender. Bania, K. (2026). https://BioRender.com/7tcsqj7.

Conventional MALDI matrices and emerging antioxidant-derived matrix candidates are compared schematically in Figure 3. Established matrices such as CHCA, DHB, and SA have well-characterized analytical applications but may be associated with limitations, including low-*m*/*z* background signals and crystallization-dependent variability. Importantly, conventional and antioxidant-derived matrix categories are not mutually exclusive, as established matrices such as DHB may also exhibit antioxidant properties. Selected natural antioxidants are being investigated as potential alternative matrix materials; however, their analytical performance remains dependent on compound structure, analyte properties, and experimental conditions and requires direct experimental validation.

**Table 1:** Comparison of the core properties, applications, limitations, and representative performance metrics of common conventional MALDI matrices.

Matrix	Chemical Name	Primary Analyte Class(es)	Key Advantages	Key Limitations	Representative Performance Metrics	Typical Application Focus	References
CHCA	α-Cyano-4-hydroxycinnamic acid	Peptides, small proteins	High ionization efficiency, forms homogeneous small crystals, excellent sensitivity	High chemical background in the low-*m*/*z* region (<500), can cause analyte fragmentation	Quantitative performance is strongly preparation- and analyte-dependent. In comparative studies, CHCA showed good reproducibility, while micro-spotting improved sample homogeneity for CHCA, DHB, and SA.	Proteomics, peptide mass fingerprinting	[4,5,20,23,24]
DHB	2,5-Dihydroxybenzoic acid	Peptides, lipids, glycans, oligosaccharides, natural products	“Cooler” matrix (less fragmentation), broad applicability, good for labile molecules	Forms large, heterogeneous crystals (“sweet spots”), poor shot-to-shot reproducibility	In quantitative MALDI-FTICR-MS of protease inhibitors, mean quantitative precision ranged from 6.4–11.1% RSD, quantitative accuracy from 4.5–8.0% deviation, and LOQs from 4–100 fmol/µL under the reported conditions.	Lipidomics, glycomics, small-molecule analysis	[4,5,15,20,21,25]
SA	Sinapinic Acid	Intact proteins, high-molecular-weight polymers	Promotes intact molecular ions, reduced fragmentation for large analytes	Low sensitivity for small molecules, high background in low-*m*/*z* region	Matrix choice and preparation influence repeatability, reproducibility, signal strength, background intensity, resolution, and detectability. Comparative studies have evaluated SA alongside CHCA and DHB, but standardized matrix-independent performance values are not available.	Top-down proteomics, intact protein profiling	[4,12,15,23,24]

## 3. Antioxidants: Classification and Chemical Characteristics

### 3.1. Natural Antioxidants

Natural antioxidants comprise a chemically diverse group of compounds occurring predominantly in plants and plant-derived foods. Their antioxidant properties arise from different molecular structures and mechanisms. Among the major classes, polyphenols—including phenolic acids, flavonoids, stilbenes, lignans, and tannins—represent one of the most extensively studied groups [26,27,28]. They are widely distributed in fruits, vegetables, cereals, legumes, tea, coffee, nuts, and other plant materials, contributing substantially to the antioxidant composition of many foods [26,29]. From an analytical perspective, their structural diversity is particularly important because differences in hydroxylation, methoxylation, glycosylation, polymerization, and degree of conjugation can markedly influence the physicochemical properties of individual compounds [27,28].

Flavonoids constitute a major subgroup of plant polyphenols and share a common C6–C3–C6 carbon framework. Structural modifications of this framework give rise to several subclasses, including flavonols, flavones, flavanones, flavan-3-ols, anthocyanins, and isoflavonoids. Compounds such as quercetin, kaempferol, catechin, epicatechin, and anthocyanins are widely present in plant foods and have been extensively studied because of their antioxidant and other biological activities. Their structures contain aromatic rings and multiple functional groups capable of participating in redox reactions, while differences in substitution patterns influence their antioxidant behavior and interactions with surrounding matrix components [27,30]. Phenolic acids represent another important group and are commonly divided into hydroxybenzoic and hydroxycinnamic acids according to their basic carbon skeleton. Gallic, protocatechuic, caffeic, ferulic, and *p*-coumaric acids are frequently found in plant-derived materials, either in free form or associated with other cellular components [26,28,31]. The distinction between extractable and non-extractable phenolics is also analytically relevant, as a substantial fraction of phenolic compounds may remain associated with cell-wall components or occur in polymeric forms [29].

Tannins represent a structurally heterogeneous group of polyphenols and are broadly classified as hydrolyzable or condensed tannins. Their relatively large and complex structures distinguish them from simpler phenolic compounds and influence both their interactions with proteins and other macromolecules and their behavior during extraction and analysis. Condensed tannins are widely distributed in plant foods and may undergo significant changes during food processing, thereby affecting their measurable antioxidant properties [32]. Lignans and related phenolic compounds constitute additional classes of naturally occurring antioxidants and are present in various seeds, cereals, fruits, and other plant-derived materials [26,33]. Together, these compounds illustrate the structural diversity that must be considered when developing analytical approaches for natural antioxidants.

Natural antioxidants are not limited to phenolic compounds. Carotenoids constitute an important class of lipid-soluble pigments characterized by extended systems of conjugated double bonds. β-Carotene, lycopene, lutein, and astaxanthin are among the carotenoids most commonly investigated in relation to antioxidant activity. Their chemical properties differ substantially from those of phenolic antioxidants, particularly with respect to polarity and lipid solubility, and these differences influence both their distribution in biological and food matrices and their analytical behavior [34,35]. Antioxidant vitamins represent another chemically distinct group. Ascorbic acid is a water-soluble antioxidant involved in redox reactions and cellular antioxidant defense, whereas vitamin E compounds, particularly tocopherols and tocotrienols, are lipid-soluble antioxidants associated primarily with lipid-containing environments [36,37]. The coexistence of water-soluble, lipid-soluble, low-molecular-weight, and polymeric antioxidants within the same biological or food sample therefore creates considerable chemical complexity for analytical characterization.

From an analytical perspective, antioxidant activity cannot be regarded as a uniform chemical property shared by all antioxidant classes. Molecular structure, polarity, degree of conjugation, functional-group composition, molecular size, and interactions with surrounding matrix components all influence the behavior of individual compounds during extraction, separation, ionization, and detection [26,28,29]. This is particularly relevant in mass spectrometric analysis, where compounds belonging to the same broad antioxidant class may exhibit substantially different ionization efficiencies. Polyphenolic antioxidants, for example, contain aromatic and hydroxylated structures that can facilitate their detection by mass spectrometric methods, whereas carotenoids possess highly conjugated, nonpolar structures that require different analytical considerations [34,35]. Similarly, antioxidants occurring as glycosides, esters, polymers, or non-extractable complexes may produce analytical profiles that differ from those of their corresponding free forms [27,29].

The chemical diversity of natural antioxidants therefore represents both a challenge and an opportunity for MALDI-MS. Compounds containing aromatic conjugated systems and functional groups capable of participating in proton-transfer or electron-transfer processes may possess physicochemical properties relevant not only to their detection as analytes but also to their possible use as matrix compounds. This is particularly important when evaluating natural antioxidants as alternative MALDI matrices, because the same structural features that determine their optical and chemical properties may also influence laser absorption, desorption, and ion formation.

**Figure 4:**
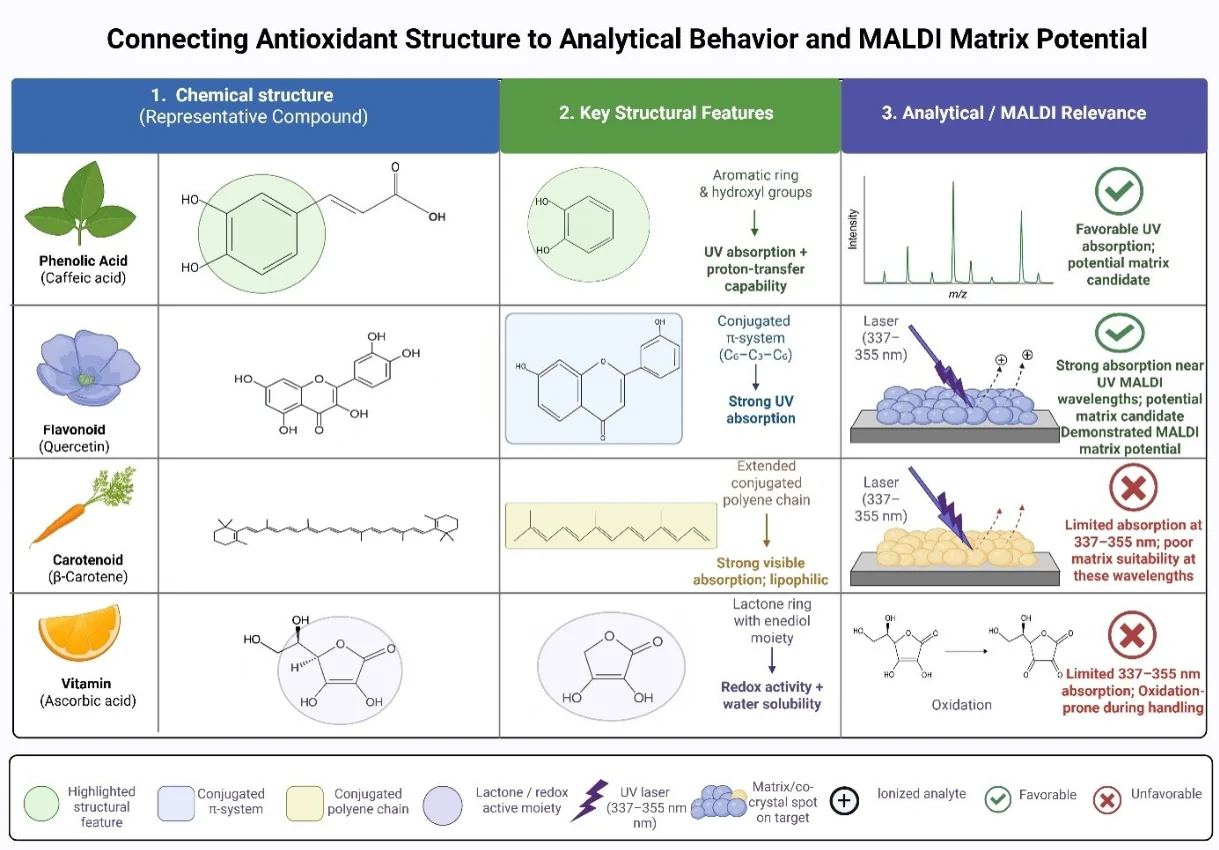
Structural features and MALDI-relevant properties of selected natural antioxidants. Caffeic acid, quercetin, β-carotene, and ascorbic acid illustrate differences in aromaticity, conjugation, polarity, and functional-group composition that may influence UV absorption, ionization behavior, and potential suitability as MALDI matrix candidates. Created in BioRender. Bania, K. (2026) https://BioRender.com/g4qicp9.

The structural diversity of selected natural antioxidants is illustrated in Figure 4. Caffeic acid and quercetin contain aromatic and conjugated systems together with hydroxyl groups that influence their optical and chemical properties. β-Carotene represents a highly conjugated, nonpolar antioxidant whose absorption properties differ substantially from those of aromatic phenolic compounds. In contrast, ascorbic acid is a non-aromatic, water-soluble antioxidant characterized by a lactone ring and enediol moiety associated with its redox activity. These structural differences influence properties relevant to MALDI-MS, including UV absorption, polarity, ionization behavior, and potential matrix suitability.

### 3.2. Vitamins and Related Antioxidants

In addition to polyphenols and carotenoids, several vitamins and vitamin-related compounds contribute to the antioxidant capacity of biological systems and foods. Among these, ascorbic acid and vitamin E represent two chemically distinct groups whose physicochemical properties strongly influence their biological distribution and analytical behavior [36,38,39]. Ascorbic acid is a water-soluble compound that readily participates in oxidation–reduction reactions and can function as an electron donor, whereas vitamin E comprises a family of lipid-soluble tocopherols and tocotrienols associated primarily with lipid-containing environments [36,38]. Their markedly different polarity and chemical structures illustrate an important consideration in antioxidant analysis: compounds grouped according to biological function may nevertheless require substantially different analytical approaches.

Ascorbic acid (vitamin C) is a relatively small molecule characterized by multiple hydroxyl groups and a lactone moiety that enables reversible oxidation to dehydroascorbic acid. Its antioxidant activity is closely related to electron-transfer reactions, while its water solubility facilitates distribution in aqueous biological compartments and food matrices [36,40]. However, the same redox reactivity that makes ascorbic acid an effective antioxidant can complicate its accurate analytical determination because oxidation may occur during sample handling and storage. Consequently, measured values may be influenced not only by the analytical technique but also by sample preparation and storage conditions [40,41]. Chromatographic and mass spectrometric methods have therefore been applied to distinguish ascorbic acid from its oxidation products and to characterize vitamin C in complex biological and food matrices [41,42].

Vitamin E comprises α-, β-, γ-, and δ-tocopherols together with the corresponding tocotrienols. Tocopherols contain a chromanol ring with a hydroxyl group capable of hydrogen donation and a hydrophobic side chain that promotes association with lipid phases and biological membranes [38,43]. Tocotrienols share the same chromanol structure as tocopherols but contain an unsaturated isoprenoid side chain, resulting in distinct molecular conformations and physicochemical properties [43,44]. Among vitamin E forms, α-tocopherol has received particular attention because of its biological significance; however, the remaining homologues also exhibit antioxidant activity and are important targets in foods and biological samples [38,43]. Their pronounced lipophilicity distinguishes them analytically from water-soluble antioxidants and requires extraction and detection strategies compatible with nonpolar or lipid-rich matrices [43].

The antioxidant activity of vitamin E compounds derives primarily from the phenolic hydroxyl group of the chromanol ring, which can participate in hydrogen-atom transfer reactions with lipid-derived radicals [38,43]. Structural differences among tocopherols and tocotrienols can influence their reactivity and interactions with lipid environments [43,44]. Their low polarity and structural similarity, however, can complicate chromatographic separation and identification, particularly when several homologues are present. Liquid chromatography-mass spectrometry has therefore become an important tool for characterizing vitamin E forms in complex samples when sensitive detection and structural differentiation are required [45].

Other natural redox-active compounds, including coenzyme Q, glutathione, and several endogenous metabolites, also contribute to antioxidant status [46,47]. Glutathione is a low-molecular-weight thiol-containing tripeptide whose reduced form plays an important role in cellular redox regulation and detoxification pathways [48,49]. These compounds are structurally and chemically distinct from both vitamins and polyphenols, emphasizing that antioxidant activity encompasses a broad range of molecular mechanisms rather than a single structural feature.

This structural diversity is particularly relevant to mass spectrometric analysis. Molecular size, ionizable groups, polarity, redox activity, and susceptibility to oxidation can influence both ion formation and ion stability [41,48]. These properties help explain why some antioxidants are readily detected by MALDI-MS, whereas others may show substantial background interference, poor co-crystallization, or inefficient ionization. The analytical behavior of vitamins and related antioxidants should therefore be considered in relation to their individual structures rather than their antioxidant status alone. When naturally occurring antioxidants are evaluated as potential MALDI matrices, features such as aromaticity, conjugation, proton-donating capacity, and UV absorption become particularly important because they may influence desorption and ionization behavior.

### 3.3. Analytical Challenges in Antioxidant Detection

The analysis of natural antioxidants is challenging because of their considerable structural and physicochemical diversity. Polyphenols, carotenoids, vitamins, and other redox-active compounds differ markedly in polarity, molecular mass, stability, and ionization characteristics, requiring analytical protocols adapted to the physicochemical properties of the target compounds [36,50,51]. This complexity is particularly relevant in biological and food matrices, where antioxidants may occur simultaneously as free compounds, glycosides, esters, oxidative transformation products, or polymeric conjugates [29,50].

Sample preparation represents an important source of variability in antioxidant analysis. Extraction efficiency depends on solvent composition, polarity, pH, temperature, extraction time, and analyte stability [29,51]. Some compounds, such as ascorbic acid, are highly susceptible to oxidation, whereas carotenoids and vitamin E compounds may degrade upon exposure to light, oxygen, or inappropriate handling conditions [36]. Differences in extraction and storage procedures can therefore alter the measured antioxidant profile independently of the analytical instrument itself [51].

Chromatographic methods, particularly HPLC and LC-MS, remain widely used because they enable the separation of sample components prior to detection and can therefore distinguish structurally related antioxidants in complex mixtures. However, these methods may require extensive sample preparation and careful method development, while closely related analytes can remain difficult to distinguish without reference standards or additional structural information [50,52]. NMR spectroscopy provides valuable structural information and supports the characterization of complex mixtures, although its sensitivity is generally lower than that of mass spectrometric techniques, limiting its applicability to some low-abundance compounds [53]. GC-MS can provide detailed structural information for sufficiently volatile compounds or analytes amenable to derivatization; however, the additional derivatization step limits its applicability across all antioxidant classes [52].

Mass spectrometric approaches provide high sensitivity and enable the detection of chemically diverse compounds while supplying molecular mass information and, when combined with tandem MS, additional structural information [50,52]. Nevertheless, ionization efficiency can vary substantially among compounds and may be influenced by matrix effects, analyte concentration, and the presence of co-eluting or co-crystallizing species [20,52]. These effects are particularly relevant in MALDI-MS, where matrix composition and physical properties directly influence analyte desorption and ion formation. For low-molecular-weight antioxidants, matrix-derived signals may overlap with analyte peaks and complicate spectral interpretation [4,20]. Reliable MALDI-MS analysis therefore requires appropriate matrix selection and careful control of background interference.

These analytical limitations have encouraged the development of alternative matrix systems designed to efficiently absorb laser energy and support ionization while minimizing background signals in the low-*m*/*z* region [4,20]. The structural properties of selected natural antioxidants make them relevant candidates for further investigation, both as target analytes and as potential alternative MALDI matrices.

## 4. MALDI-MS for the Analysis of Antioxidant Compounds

### 4.1. MALDI-MS Analysis of Individual Antioxidant Compounds

The potential use of natural antioxidants as MALDI matrices is determined primarily by their chemical and photophysical properties rather than by antioxidant activity alone. An effective MALDI matrix should absorb the laser wavelength used for ionization, support analyte desorption and ion formation, and generate sufficiently low chemical background for reliable detection [4,10,15]. Many naturally occurring antioxidants, particularly polyphenols, contain aromatic and conjugated systems that contribute to ultraviolet absorption, while hydroxyl and other ionizable groups may participate in proton-transfer and related ionization processes [10,27]. These features make selected antioxidant compounds plausible candidates for MALDI matrix applications.

Natural antioxidants encompass a broad range of structures and physicochemical properties. Phenolic acids, flavonoids, and related compounds differ in hydroxyl-group number and position, degree of conjugation, molecular weight, and acidity [27,30]. These differences are important because matrix performance depends not only on optical absorption but also on crystallization, analyte incorporation, proton-transfer behavior, and the tendency of the matrix to generate interfering ions [4,15]. Consequently, antioxidant activity alone should not be used as a selection criterion. Optical absorption, ionization behavior, crystallization characteristics, stability, and background spectrum should all be evaluated experimentally.

Another important consideration is the reduction in low-*m*/*z* background associated with conventional organic matrices. Matrix-derived ions can obscure low-molecular-weight compounds, including many antioxidants, which has encouraged the development of alternative matrix systems for small-molecule analysis [4,20]. Natural compounds may provide useful alternatives, but their suitability must be demonstrated experimentally because limited solubility, heterogeneous crystallization, structural complexity, and intrinsic ion formation may also impair MALDI performance [4,15,20].

Inorganic and nanostructured materials have also been investigated as alternative matrix or substrate materials for small-molecule LDI and MALDI. For example, titanium dioxide microparticles and nanoparticles, porous silicon, and other nanostructured substrates have been explored for low-molecular-weight analysis, with some approaches showing reduced matrix-related interference in the low-*m*/*z* region [53,54,55]. Therefore, these systems represent an important complementary strategy for addressing the background limitations of conventional organic matrices. Nevertheless, they should be distinguished from antioxidant-derived matrices because their analytical performance arises from the physiochemical properties of the inorganic or nanostructured material rather than from antioxidant functionality.

From a methodological perspective, antioxidant-derived matrix candidates should therefore be assessed according to several complementary criteria: appropriate absorption at the laser wavelength, efficient analyte desorption and ionization, favorable co-crystallization and sample homogeneity, limited matrix-derived background within the relevant *m*/*z* range, and sufficient chemical and physical stability during sample preparation and laser irradiation [4,10,15]. These criteria provide a practical framework for evaluating natural antioxidants as potential MALDI matrices.

### 4.2. Analysis of Antioxidants in Plant Extracts

Plant extracts are complex mixtures of phenolic acids, flavonoids, anthocyanins, tannins, and other secondary metabolites, which complicates comprehensive characterization when multiple structural variants are present simultaneously. MALDI-MS has been used as a complementary approach to chromatographic-mass spectrometric techniques because it enables the rapid molecular profiling and detection of structurally related compounds. An early study demonstrated the direct analysis of flavonol glycosides in food-derived extracts, where MALDI-TOF MS detected several quercetin and kaempferol glycosides and provided information about their glycosidic residues through characteristic fragmentation patterns [56,57]. This work provided early evidence for the applicability of MALDI to plant-derived antioxidant compounds.

MALDI-MS has subsequently been applied to several classes of plant antioxidants. Anthocyanins have been analyzed directly in *Arabidopsis thaliana* leaf tissues using MALDI-TOF MS, demonstrating reproducible detection without extensive chromatographic separation [58]. MALDI-TOF MS has also been used to characterize condensed tannins, whose structural heterogeneity makes their analysis particularly demanding. In *Delonix regia* extracts, MALDI-TOF MS combined with NMR and HPLC-ESI-MS revealed differences in tannin composition and polymerization among leaves, fruits, and stem bark, illustrating the complementary value of MALDI and chromatographic techniques [59].

More recent studies have extended MALDI-based approaches to the spatial analysis of antioxidant-rich plant materials. In *Gliricidia sepium*, MALDI-MS and MALDI-MSI enabled the detection of kaempferol, isorhamnetin, and fatty acids on the leaf surface and subsequent mapping of their spatial distribution [60]. MALDI-MSI has also been used in *Scutellaria baicalensis* to characterize the distribution of flavonoids and other bioactive metabolites across different tissues [61]. In *Tetrastigma hemsleyanum*, MALDI-MSI was integrated with transcriptomic and proteomic data to investigate tissue-specific flavonoid distribution and associated biosynthetic pathways [62].

An important advantage of MALDI in plant analysis is its ability to provide rapid molecular profiles with limited chromatographic separation and, in imaging applications, to preserve information about metabolite localization. This is particularly useful for antioxidants that are unevenly distributed among plant organs or within individual tissues [60,61,62]. However, the complexity of plant extracts can complicate interpretation through overlapping ions, glycosylation, adduct formation, in-source fragmentation, and variable ionization efficiency [57,59]. Therefore, compound assignments based on MALDI-MS are generally strengthened by complementary MS/MS, chromatographic, or spectroscopic evidence rather than accurate mass alone.

Matrix selection and extract composition also strongly influence the resulting spectra. In the analysis of *G. sepium*, MALDI conditions and different matrices required optimization before reliable detection of selected metabolites was achieved [60]. The direct MALDI analysis of crude plant extracts should therefore be regarded primarily as a profiling approach, because sample preparation and matrix selection may influence both the detection and relative intensity of antioxidant signals. Its main advantages are rapid molecular screening and spatial mapping, whereas unambiguous structural identification and quantitative analysis generally require complementary analytical techniques.

### 4.3. Food Analysis

Food matrices contain chemically diverse antioxidants whose concentrations and molecular forms may be altered by processing, storage, and interactions with other food components. MALDI-MS has been applied mainly to the profiling of flavonoids, anthocyanins, catechins, and related polyphenols. Early studies showed that MALDI-TOF MS could identify flavonol glycosides directly in food extracts through characteristic molecular and fragment ions derived from quercetin and kaempferol glycosides [57]. MALDI-TOF MS has also been used for rapid profiling of soy isoflavones, including daidzin and genistin, and for detecting changes associated with food processing [63]. Although these studies employed conventional organic matrices, they demonstrated the feasibility of the MALDI-based analysis of low-molecular-weight antioxidants in complex food samples.

Anthocyanin-rich foods have also been investigated using MALDI-MS. Studies of red wine and fruit juices demonstrated the detection of intact anthocyanin molecular ions and enabled the qualitative comparison of anthocyanin profiles among different fruit sources [64]. This is relevant to pigmented foods, where numerous structurally related anthocyanins may coexist in glycosylated and acylated forms. MALDI-MS has additionally been applied to grape- and wine-derived polyphenols, including procyanidin oligomers, demonstrating its utility for structurally complex and relatively high-molecular-weight antioxidant species [65].

Tea represents a particularly complex food matrix because processing generates mixtures of catechins, oxidized catechin derivatives, theaflavins, and thearubigins. The MALDI-TOF MS analysis of thearubigin fractions from black tea produced heterogeneous signals across a broad mass range and also demonstrated a relevant analytical limitation: highly redox-active polyphenols and matrix compounds may undergo oxidation and contribute to analytical artifacts [66]. Subsequent studies combining MALDI-TOF MS with complementary mass spectrometric techniques further characterized oxidized catechin oligomers and emphasized the value of MS/MS and chromatographic separation for highly complex samples [67].

MALDI-based methods have also been combined with chromatographic and planar separation techniques. Direct HPTLC-MALDI-TOF MS has been applied to flavonoid analysis in phytochemical fingerprints, allowing mass spectrometric information to complement chromatographic separation [68]. Similarly, TLC-MALDI-TOF MS has been used for flavonoid dereplication in samples including wine, apple, and rose, demonstrating that prior separation can reduce sample complexity before mass analysis [69]. Such approaches are particularly useful when structurally related compounds have similar masses or direct MALDI spectra contain overlapping signals.

MALDI-MSI has further expanded food analysis by enabling the spatial characterization of antioxidant compounds within intact samples. The technique has been applied to visualize both polar anthocyanins and nonpolar β-carotene in fresh and processed foods, demonstrating that chemically distinct antioxidants can be investigated in their original spatial context [70]. Studies of polyphenol imaging have similarly highlighted MALDI-MSI as a useful approach for examining the distribution of compounds in tea, coffee, fruits, and vegetables rather than relying exclusively on bulk measurements after extraction [71]. Such spatial information may help reveal how processing and tissue organization influence antioxidant distribution.

Overall, MALDI-MS provides complementary approaches for the characterization of food antioxidants, ranging from rapid molecular profiling to the analysis of oligomeric polyphenols and spatial mapping by MALDI-MSI. Its application to complex food matrices remains limited by overlapping signals, oxidation, adduct formation, matrix effects, and variable ionization efficiency [66,67]. MALDI-MS is therefore most informative when combined with LC-MS, MS/MS, or other orthogonal techniques rather than used as a standalone method. Importantly, its successful application to chemically diverse antioxidants also provides an experimental basis for investigating selected natural compounds as potential alternative MALDI matrices.

### 4.4. Biological Samples

The analysis of antioxidants in biological samples is more challenging than the analysis of purified compounds or relatively simple plant extracts. Antioxidants occur at different concentrations and in multiple chemical forms, while proteins, lipids, salts, and other endogenous metabolites may interfere with extraction and ionization. Consequently, MALDI-based methods are used primarily for molecular profiling and spatial characterization rather than as routine standalone techniques for quantitative antioxidant determination.

MALDI-MS has been applied to biological samples containing antioxidant-related metabolites, particularly low-molecular-weight polyphenols and their metabolic derivatives. Because these compounds may undergo glucuronidation, sulfation, methylation, and other transformations in vivo, the analysis of the parent compound alone may not adequately represent their metabolic fate [72,73]. Mass spectrometry is particularly useful in this context because information from molecular mass and fragmentation patterns can help distinguish parent compounds from structurally modified metabolites [72,74].

MALDI-MSI provides an additional means of investigating antioxidant-related compounds directly in tissue sections while preserving spatial information that is lost during homogenization. This approach has been used to examine plant-derived polyphenols and their localization in animal tissues after exposure, although detection remains dependent on compound abundance, tissue composition, extraction efficiency, and matrix selection [74,75]. MALDI-MSI has also been incorporated into metabolomic studies of spatial changes in endogenous metabolites associated with disease, treatment, and metabolic alterations [75,76].

A major challenge in biological antioxidant analysis is the relatively low abundance of many target compounds compared with endogenous lipids, peptides, and other metabolites. Matrix effects and ion suppression can substantially influence signal intensity, while salts and other biological components may promote adduct formation and complicate spectral interpretation [20,76]. These problems are particularly relevant for low-molecular-weight antioxidants because conventional organic matrices may produce intense signals in the low-*m*/*z* region that overlap with analytes of interest [4,20].

MALDI-MS is therefore currently most useful for the targeted or exploratory profiling of antioxidant-related compounds in biological matrices, while MALDI-MSI additionally enables investigation of their spatial distribution, rather than either approach serving as a universal method for quantitative antioxidant determination. Reliable identification and quantification require careful sample preparation, appropriate matrix selection, suitable controls, and complementary LC-MS or MS/MS measurements [4,20,72]. These limitations further support the investigation of alternative matrix systems capable of reducing low-*m*/*z* background and improving the detection of small antioxidant compounds, including the antioxidant-derived matrix candidates discussed in Section 6.

### 4.5. MALDI Imaging of Antioxidants

MALDI-MSI extends the antioxidant analysis by providing spatial information on metabolite distribution directly within tissues. However, MALDI-MSI should be clearly distinguished analytically from conventional MALDI-MS because the reliability of an imaging experiment depends on molecular detection and ionization as well as the preservation and interpretation of spatial information. Additional variables (e.g., spatial resolution, matrix deposition, analyte delocalization, tissue heterogeneity, matrix crystal size and coating characteristics, ion suppression, and spatial registration) must therefore be considered when interpreting MSI data. This is particularly relevant for plant-derived antioxidants because flavonoids, phenolic acids, anthocyanins, and other secondary metabolites are often heterogeneously distributed among tissues and cellular compartments. Recent studies have shown that MALDI-MSI can simultaneously profile multiple classes of plant metabolites and associate their signals with specific anatomical structures [61,62].

Flavonoids represent one of the more established applications of MALDI-MSI in antioxidant research. In *Scutellaria baicalensis*, MALDI-MSI was used to characterize the distribution of flavonoids and related metabolites across roots, stems, and leaves [61]. Similarly, in *Tetrastigma hemsleyanum*, MALDI-MSI combined with transcriptomic and proteomic data enabled the investigation of tissue-specific flavonoid distribution and associated metabolic pathways [62]. These studies illustrate an important advantage of MALDI-MSI over bulk extraction methods: compounds with similar overall abundance may exhibit markedly different spatial distributions that are lost during sample homogenization.

MALDI-MSI has also been applied to antioxidant compounds in food matrices. The spatially resolved analysis of polyphenols and other metabolites in fruits, vegetables, tea, and processed foods has demonstrated that localization information can be preserved without relying exclusively on bulk extraction [70,71]. Imaging has been particularly useful for anthocyanins and flavonoids in pigmented tissues, where metabolite distribution can be related to tissue architecture and processing [70]. This approach may therefore provide information on how food structure and processing influence the spatial distribution of antioxidant compounds.

Applications have also been extended to antioxidant-related compounds in animal tissues. Following the exposure to dietary polyphenols or other bioactive compounds, MALDI-MSI may enable the spatial differentiation of parent compounds, metabolic derivatives, and endogenous metabolites, provided that the analyte concentrations are sufficient for detection and that appropriate standards or complementary analytical methods are available [74,75]. This capability is particularly valuable when tissue localization is an important component of the biological question.

Despite these advantages, the MALDI-MSI analysis of antioxidants remains technically demanding. Ionization efficiency may differ substantially among chemically related compounds, while matrix application, tissue composition, analyte delocalization, and ion suppression can influence both signal intensity and apparent spatial distribution [4,20,75].

In addition, spatial resolution and the dimensions and uniformity of matrix crystals or coatings can affect the spatial information obtained, while spatial registration with the corresponding tissue or sample morphology is required for reliable spatial interpretation. Low-molecular-weight antioxidants present an additional challenge because conventional organic matrices may generate abundant background ions in the same *m*/*z* region [4,20]. Notably, the detection of a spatially resolved *m*/*z* feature should be distinguished from validated molecular identification. Tentative molecular assignment may be supported by an *m*/*z* signal, but spatial localization itself does not establish chemical identity. Spatially resolved signals should therefore not be regarded as definitive compound identification without supporting evidence from accurate mass measurements, MS/MS, chromatography, reference standards, or other orthogonal methods.

Overall, MALDI-MSI provides a valuable extension of MALDI-MS by combining molecular detection with spatial information. Current applications demonstrate its usefulness for mapping flavonoids and other antioxidant-related metabolites in plants and foods, while studies in animal tissues offer opportunities to investigate the localization of dietary antioxidants and their metabolites. Further improvements in matrix chemistry, ionization efficiency, spatial resolution, and compound identification are still required, particularly for low-molecular-weight antioxidants.

**Figure 5:**
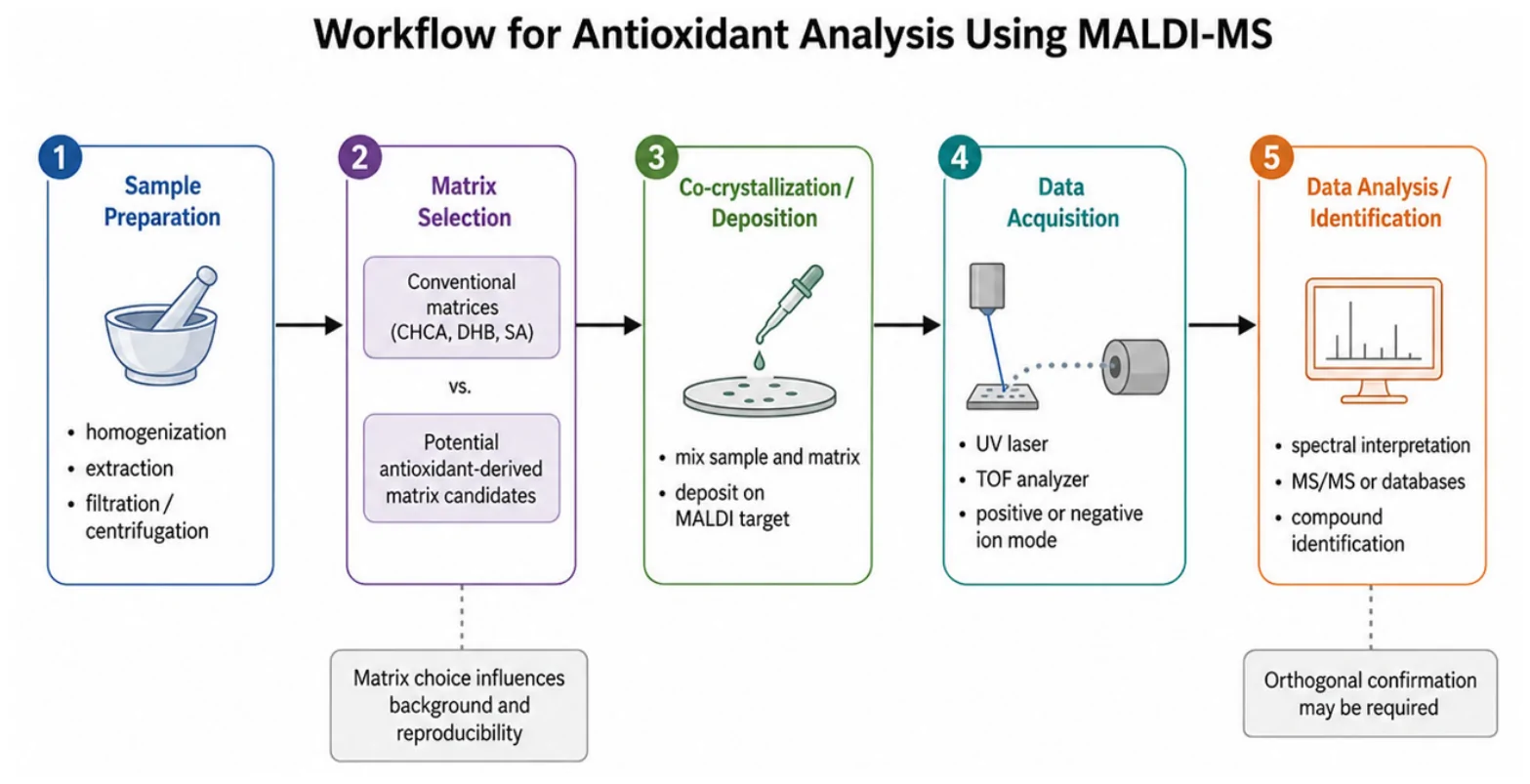
General workflow for MALDI-MS analysis of antioxidant compounds. The main stages include sample preparation, matrix selection, matrix–analyte deposition, data acquisition, and compound identification. Conventional matrices and potential antioxidant-derived matrix candidates are distinguished because matrix selection can influence background interference, ionization efficiency, and analytical reproducibility. Orthogonal confirmation may be required for reliable compound identification. Created in BioRender. Bania, K. (2026) https://BioRender.com/ucp71v1.

The general workflow for MALDI-MS analysis of antioxidant compounds is summarized in Figure 5. It includes sample preparation, matrix selection, matrix–analyte deposition, data acquisition, and subsequent spectral interpretation and compound identification. Matrix selection represents a particularly important step because conventional matrices and potential antioxidant-derived matrix candidates may differ in background generation, crystallization behavior, and ionization performance. Reliable compound assignment may additionally require MS/MS, database comparison, reference standards, or other complementary analytical approaches.

## 5. Natural Antioxidants as Alternative MALDI Matrices

### 5.1. Why Antioxidants Can Function as MALDI Matrices

The rationale for investigating antioxidant-derived MALDI matrices arises from the overlap between some structural features associated with antioxidant compounds and those required for effective matrix-assisted laser desorption/ionization. An effective matrix should absorb laser radiation and facilitate energy or charge transfer during analyte desorption and ionization. This requirement is particularly important for low-molecular-weight analytes because conventional organic matrices may generate abundant background ions in the low-*m*/*z* region and interfere with target signals [20]. Consequently, alternative matrix development has increasingly focused on materials capable of supporting ionization while reducing spectral interference.

In this review, the term “antioxidant-derived MALDI matrix” is used as the general term for a material originating from a naturally occurring antioxidant compound or antioxidant-containing material that is evaluated as an MALDI matrix. This term refers to defined low-molecular-weight antioxidant molecules, naturally derived antioxidant-rich materials such as lignin, and hybrid or composite systems containing an antioxidant-derived component. The distinction between these categories is based on their chemical and material form and is not considered chemically equivalent. The terms “natural matrix” and “natural antioxidant matrix” are avoided as general descriptors because they do not specify whether the material is a defined antioxidant compound, a complex natural material, or a composite system. Similarly, “phenolic matrix” is used only when referring specifically to the phenolic chemical character of a matrix and is not treated as a synonym for antioxidant-derived MALDI matrix.

Polyphenolic and other naturally occurring antioxidant structures are of particular interest because they frequently contain aromatic or conjugated systems together with phenolic hydroxyl and other functional groups. These characteristics influence optical properties, acidity, hydrogen bonding, and redox behavior [77,78]. Lignin provides an instructive example because its aromatic and phenolic framework is associated with antioxidant activity, while its relatively high molecular weight may reduce the formation of low-mass matrix-derived ions [77,79]. However, the lignin structure and antioxidant properties vary with molecular weight, phenolic hydroxyl content, degree of condensation, botanical origin, and processing conditions [78,79]. Such variability may also influence its performance as an MALDI matrix.

Experimental evidence supports this concept for at least some naturally derived materials. Zhao et al. demonstrated that lignin can function as a dual-polarity MALDI matrix for small molecules, enabling qualitative detection of 30 compounds and quantitative determination of selected analytes with good linearity. The method was also applied to myricitrin in Chinese bayberry, where MALDI-based results showed close agreement with HPLC measurements across different cultivars and tissues. Spectroscopic characterization was used to investigate the structural properties potentially associated with lignin matrix performance [80].

Lignin is particularly relevant to the concept of antioxidant-derived matrices because its phenolic moieties contribute to antioxidant activity, whereas its extended aromatic structure influences its physicochemical behavior [77,78,79]. This distinction is important: matrix suitability should not be attributed to radical-scavenging activity alone. Instead, antioxidant activity may coexist with structural properties that influence laser absorption, molecular interactions, charge transfer, and desorption. Each of these properties should therefore be evaluated independently when assessing a potential matrix.

Redox chemistry may also contribute to matrix behavior. MALDI ionization mechanisms depend on both matrix and analyte properties and may involve proton-transfer and electron-transfer pathways. Recent approaches to matrix design have therefore considered electronic properties as predictors of ionization behavior [4,81]. This is relevant to natural antioxidants because many contain oxidizable phenolic groups and conjugated systems. Nevertheless, the reducing capacity alone cannot be regarded as evidence of matrix suitability. The relevant question is whether the electronic properties of a specific compound support the ionization pathway required for a given analyte under defined experimental conditions.

Naturally derived materials may also be compatible with strategies aimed at reducing the matrix-derived background. High-molecular-weight, polymeric, hybrid, and nanostructured matrices have demonstrated that matrix size and physical architecture can influence background generation while maintaining useful optical and ionization properties [20,82]. Lignin is particularly relevant in this context because its extended aromatic framework and phenolic functionality distinguish it from conventional low-molecular-weight organic matrices [80]. Other emerging matrix systems, including metal–organic and hydrogen-bonded organic frameworks, similarly demonstrate that matrix–analyte interactions can be engineered to improve background suppression, salt tolerance, and signal reproducibility for small-molecule analysis [82].

The available evidence, however, does not support antioxidant activity as an independent matrix-selection criterion. Natural antioxidants differ markedly in molecular weight, solubility, crystallization behavior, UV absorption, acidity, redox potential, and chemical stability [77,78,79]. Naturally derived materials such as lignin may also show substantial batch-to-batch heterogeneity resulting from differences in botanical origin and extraction or processing procedures [78,79]. Such variability represents an important challenge for analytical reproducibility.

Current evidence therefore provides a chemical rationale for investigating selected natural antioxidants as potential MALDI matrices, but matrix suitability must be established experimentally for each compound. Candidates combining aromatic or conjugated systems, ionizable functional groups, and favorable physical properties may provide a rationale for experimental investigation, but these features alone do not establish matrix suitability. The following sections therefore distinguish between experimentally demonstrated matrix systems and antioxidant compounds that remain primarily theoretical or chemically plausible candidates. The potential of antioxidant compounds to serve as matrices in MALDI relies on a range of structural and physicochemical characteristics that are crucial to the MALDI process. Aromatic and conjugated systems—found in many polyphenolic antioxidants such as hydroxycinnamic acids, flavonoids, and curcuminoids—enable the absorption of laser radiation and facilitate energy transfer during desorption and ionization [80].

Phenolic hydroxyl groups, carboxyl groups, and other ionizable functional groups can further participate in proton-transfer reactions, thereby influencing analyte ion formation. Redox activity, a hallmark of many antioxidants, may also contribute to electron-transfer processes, with the relative contribution of proton- and electron-transfer mechanisms likely depending on the chemical structure of both the antioxidant and the analyte. Furthermore, hydrogen bonding and other matrix-analyte interactions can influence the incorporation of the analyte into matrix crystals and its subsequent desorption. These phenomena are closely linked to the physicochemical properties of specific antioxidants and cannot be predicted solely based on their antioxidant activity [4,81].

For instance, the extensive conjugated systems and the presence of phenolic groups in caffeic, ferulic, and sinapic acids provide properties that predispose these compounds to function as MALDI matrices; in the case of compounds such as quercetin or curcumin, however, an individual assessment of parameters—such as laser radiation absorption, ionization behavior, crystallization, background signals, and compatibility with the analyte—is required [82].

Thus, the mechanism of action of antioxidant-derived MALDI matrices relies on a complex interplay of optical absorption, acid–base and redox properties, matrix–analyte interactions, and solid-phase behavior, rather than solely on antioxidant activity. Compared with conventional MALDI matrices such as α-cyano-4-hydroxycinnamic acid (CHCA) and 2,5-dihydroxybenzoic acid (DHB), antioxidant-derived matrix candidates may exhibit both overlapping and distinct physicochemical properties. Importantly, antioxidant-related properties are not exclusive to alternative matrix candidates, as established MALDI matrices such as DHB may also exhibit antioxidant activity. CHCA and DHB are well-established matrices with predictable crystallization behavior and broad applications in MALDI-MS, particularly for peptides, proteins, lipids, and other biomolecules [83]. Their effectiveness is primarily associated with efficient laser-energy absorption, matrix-assisted desorption, and proton-transfer processes. Antioxidant-derived matrix candidates may provide similar functions when they contain suitable aromatic or conjugated structures and ionizable functional groups. However, differences in molecular structure, redox activity, and physicochemical properties may influence electron-transfer processes, matrix–analyte interactions, crystallization, and background formation. The use of antioxidant compounds does not inherently provide a superior ionization performance. Compared with CHCA and DHB, many antioxidant-derived matrix candidates remain less extensively characterized and may exhibit greater variability in crystallization, analyte compatibility, and reproducibility [84]. Therefore, their potential value should be evaluated by a direct comparison with established matrices rather than inferred from antioxidant activity alone.

### 5.2. Classes of Antioxidant-Derived Matrices

In this review, the term “antioxidant-derived MALDI matrix” refers to a naturally occurring compound or naturally derived material with documented antioxidant or radical-scavenging properties that is investigated or used as an MALDI matrix because its chemical and physicochemical properties may support analyte desorption and ionization. Antioxidant activity alone, however, does not qualify a compound as an MALDI matrix. To function as a matrix, a compound must also possess properties relevant to MALDI analysis, including suitable laser absorption, acid–base or electron-transfer characteristics, matrix–analyte interactions, and appropriate crystallization and desorption behavior. Importantly, the detection of an antioxidant by MALDI-MS as an analyte does not demonstrate that the same compound can function as a matrix for other analytes.

In this review, an antioxidant-derived MALDI matrix is defined as a naturally occurring antioxidant compound or naturally derived antioxidant-rich material that has been experimentally demonstrated to facilitate the desorption and ionization of another analyte under MALDI conditions. Importantly, the ability of a compound to be detected or efficiently ionized by MALDI-MS does not, by itself, establish its function as an MALDI matrix. A matrix must provide an environment that promotes analyte desorption and ionization, through appropriate physicochemical properties such as laser absorption, crystallization behavior, proton- or electron-transfer capacity, and matrix–analyte interactions. Therefore, compounds that function primarily as MALDI analytes should not be classified as matrices unless their ability to facilitate the ionization of other compounds has been experimentally demonstrated.

Based on the available evidence, antioxidant-related compounds and materials discussed in the context of MALDI-MS can be distinguished into four groups: established antioxidant compounds that also have documented MALDI matrix applications; antioxidant-derived compounds investigated as emerging matrix candidates; naturally derived, structurally complex antioxidant-rich materials; and non-antioxidant alternative or hybrid matrix materials. This distinction is important because not all alternative MALDI matrices are antioxidants, and the presence of antioxidant activity should not be interpreted as evidence of matrix functionality.

#### 5.2.1. Established Antioxidant MALDI Matrices

Some compounds with antioxidant properties are already established or previously investigated as MALDI matrix materials. Sinapinic acid represents a particularly important example, as it is a well-established MALDI matrix and also belongs to the broader group of phenolic compounds with antioxidant properties. Caffeic acid and ferulic acid are also relevant examples of phenolic antioxidants that have been investigated in the context of MALDI matrix development. These compounds demonstrate that antioxidant activity and MALDI matrix functionality can coexist within the same molecule.

Their established or previously investigated matrix applications should therefore be distinguished from the concept of newly emerging antioxidant-derived matrices. The novelty of antioxidant-derived matrix research should not be defined simply by the antioxidant properties of a compound. Instead, attention should be directed toward compounds for which matrix functionality has not yet been sufficiently established and toward understanding how antioxidant-related chemical properties influence ionization, background formation, crystallization, and analyte selectivity.

#### 5.2.2. Emerging Antioxidant-Derived Matrix Candidates

Flavonoids comprise a structurally diverse group of plant polyphenols, including quercetin, kaempferol, catechin, and related derivatives. Their aromatic systems and multiple hydroxyl groups provide physicochemical properties relevant to MALDI ionization, and their behavior as analytes has been widely investigated [30,85,86,87,88]. However, the efficient ionization of a flavonoid as an analyte does not demonstrate its ability to facilitate ionization of another compound. Individual flavonoids should therefore be considered matrix candidates only when their matrix function has been experimentally demonstrated.

Quercetin and related flavonoids are chemically interesting because their conjugated structures and phenolic hydroxyl groups provide UV-absorbing and redox-active properties. Their hydrogen-bonding capacity may additionally influence interactions with co-crystallized analytes. These same properties, however, may affect crystallization or contribute to matrix-derived ions and fragmentation. Matrix performance is therefore expected to depend on the analyte and experimental conditions rather than antioxidant activity alone [20,88].

Phenolic acids constitute a significant group of low-molecular-weight compounds with antioxidant properties that are relevant to the MALDI-MS technique. Caffeic, ferulic, and gallic acids, as well as related phenolic acids, contain aromatic systems and hydroxyl and/or carboxyl groups capable of participating in proton transfer processes and other ionization mechanisms [89].

However, it must be emphasized that these compounds should not automatically be regarded as potential new matrices for MALDI analysis. While caffeic and ferulic acids have been investigated for use as MALDI matrices, sinapinic acid is already an established, conventional matrix in this technique. Thus, the structural similarity of these compounds to other matrices derived from cinnamic and phenolic acids provides a useful mechanistic basis for understanding matrix function; yet, it also demonstrates that employing a compound with antioxidant properties as an MALDI matrix is not a novel concept. For compounds such as gallic acid or other less common candidates with antioxidant properties, direct experimental evaluation is required to determine whether their antioxidant and physicochemical characteristics translate into effective matrix performance.

Their relatively low molecular weight, however, may present an important limitation. Matrix-derived ions and clusters can interfere with target compounds occupying the same low-*m*/*z* region, particularly when the analytes are themselves small phenolic antioxidants. The suitability of phenolic acids should therefore be evaluated not only according to laser absorption but also according to their intrinsic background spectra and interference within the analytical mass range [5,20].

Curcuminoids and Related Plant Phenolics

Curcumin represents a structurally distinct antioxidant candidate because its extended conjugated system provides substantial optical absorption, while its phenolic and diketone functionalities participate in acid–base and redox chemistry [90]. These characteristics make curcumin chemically relevant to matrix development, although its practical performance must be evaluated independently of its established antioxidant activity. More broadly, naturally occurring pigments and conjugated phenolics may represent useful sources of matrix candidates because their optical properties can be compatible with laser-driven desorption and ionization [90,91].

Other antioxidant classes, including vitamins and carotenoids, can also be considered potential antioxidant-derived matrix candidates, although their suitability is less evident. Vitamin C is highly polar and lacks the extended aromatic systems characteristic of many conventional matrices, whereas vitamin E compounds and carotenoids exhibit different combinations of hydrophobicity and conjugation [43,92,93]. These properties do not necessarily satisfy conventional matrix requirements in the same way as aromatic polyphenols. Their inclusion therefore illustrates why antioxidant compounds cannot be assumed to show uniform matrix suitability.

Overall, compounds such as quercetin, gallic acid, curcumin, catechins, and other less-established antioxidant compounds should be considered potential or emerging matrix candidates only when their matrix performance is evaluated experimentally. Their classification as antioxidants provides the chemical context for investigation but does not itself establish MALDI matrix functionality.

#### 5.2.3. Naturally Derived and Antioxidant-Rich Materials

Tannins differ from flavonoids and phenolic acids because of their oligomeric or polymeric structures containing multiple phenolic groups. Their relatively high molecular weight may reduce low-*m*/*z* interference associated with small organic matrices, while their phenolic groups provide extensive hydrogen-bonding and redox functionality [92,93,94].

Lignin represents a particularly relevant naturally derived phenolic material. Although it is not a chemically defined low-molecular-weight antioxidant and should not be equated directly with simple polyphenols, lignin possesses documented antioxidant properties and has been experimentally demonstrated as an MALDI matrix for small molecules in both positive and negative ion modes [85]. This finding provides direct proof-of-concept that a naturally derived phenolic material can function as an MALDI matrix.

The lignin study also demonstrates that matrix performance may arise from the combination of chemical functionality and physical architecture rather than antioxidant activity alone. Lignin enabled the detection of multiple small molecules and was successfully applied to myricitrin in plant samples [85]. Its performance therefore provides an experimentally supported example of a naturally derived phenolic matrix and illustrates the relevance of molecular weight and structural complexity to matrix development.

Because materials such as lignin and tannins are chemically heterogeneous, their classification should be distinguished from that of purified antioxidant compounds. Their composition, molecular structure, and physicochemical properties may vary according to origin and processing, which can influence both antioxidant properties and MALDI performance.

#### 5.2.4. Non-Antioxidant Alternative and Hybrid Matrix Materials

Not all alternative MALDI matrices discussed in the broader literature are antioxidant-derived. Nanostructured and particulate materials, including graphene-based materials, silicon nanoparticles, and metal oxides, can provide useful matrix or matrix-assisted laser desorption/ionization properties through their optical, electronic, thermal, or surface characteristics. However, within the framework of this review, these materials should not be classified as antioxidant-derived matrices unless an antioxidant component is demonstrably incorporated into the matrix system or is responsible for the relevant matrix functionality.

Such materials are therefore considered here primarily as comparative or hybrid strategies. For example, graphene oxide has been combined with conventional matrices including DHB, CHCA, THAP, and sinapinic acid to improve crystallization and analytical reproducibility [95]. Similarly, silicon nanoparticles and metal oxide materials have been investigated for small-molecule LDI applications [53,54,55]. These systems demonstrate that matrix performance can be achieved through mechanisms that are distinct from antioxidant activity.

Hybrid systems combining an antioxidant-derived compound with a nanostructured or inorganic material should likewise be interpreted cautiously. Improved performance in such systems cannot automatically be attributed to the antioxidant component alone because surface effects, enhanced energy absorption, altered crystallization, and changes in analyte adsorption may contribute to the observed analytical response.

Taken together, these categories emphasize that antioxidant status and MALDI matrix functionality represent two separate properties. Polyphenolic compounds provide a chemically plausible starting point for antioxidant-derived matrix development because aromaticity, conjugation, phenolic functionality, and redox behavior may contribute to matrix-relevant properties. Nevertheless, the strength of experimental evidence varies considerably among compounds. Lignin currently provides a clear proof-of-concept for a naturally derived phenolic matrix, whereas compounds such as quercetin, gallic acid, curcumin, and catechins require evaluation based on direct experimental evidence of matrix performance rather than antioxidant properties alone.

However, despite those factors working in their favor, experimental validation remains uneven across individual compounds. Lignin, quercetin, caffeic acid, ferulic acid, and curcumin have been experimentally demonstrated as MALDI matrix materials in studies that involve other analytes, whereas the detection of an antioxidant as an MALDI analyte alone does not establish matrix function. Gallic acid and catechins should therefore be regarded as unvalidated matrix candidates until direct experimental evidence demonstrates their ability to facilitate ionization of other analytes. The distinction and clarification between compounds discussed above is presented more clearly in Table 2.

Importantly, only compounds for which direct experimental evidence supports their function as MALDI matrices are included in Table 2. The table therefore does not include antioxidants that have been successfully detected or ionized by MALDI-MS but have not been demonstrated to facilitate the desorption and ionization of other analytes. The experimentally demonstrated materials encompass both established MALDI matrices with antioxidant-related chemical properties and naturally derived compounds that have been investigated specifically as alternative matrix materials. Other antioxidant compounds discussed in this section should be regarded as potential matrix candidates until their matrix function is experimentally validated.

**Figure 6:**
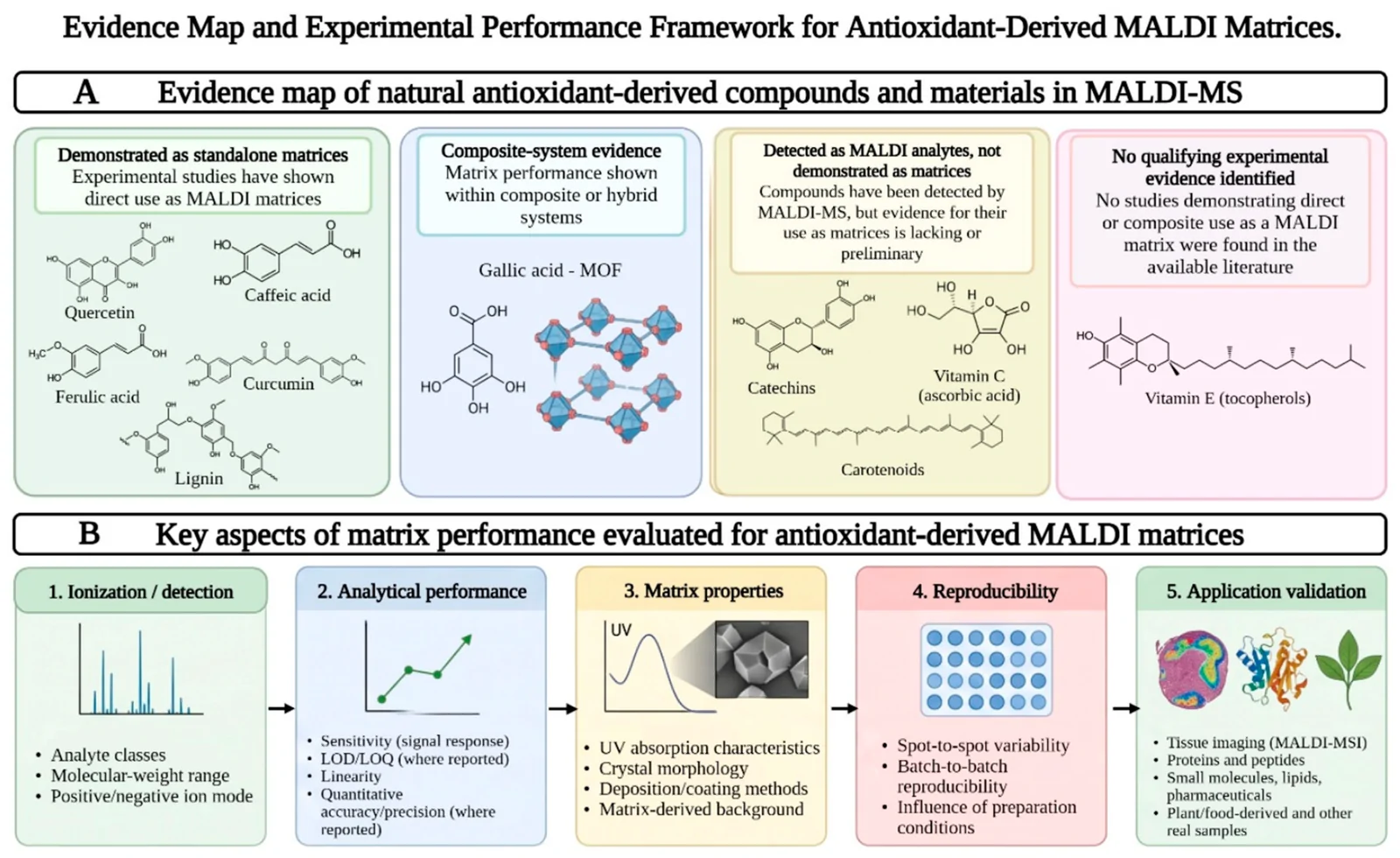
Evidence map and experimental performance framework for antioxidant-derived MALDI matrices. (**A**) Classification of antioxidant-derived candidates according to experimental evidence. (**B**) Key dimensions for evaluating matrix performance, including ionization, analytical performance, matrix properties, reproducibility, and application validation. Created in BioRender. Bania, K. (2026) https://BioRender.com/0s0ipbu.

To provide a concise overview of the current evidence and key factors influencing the performance of antioxidant-derived MALDI matrices, Figure 6 summarizes the experimentally supported matrix classes and the main analytical parameters relevant to their evaluation.

### 5.3. Matrix Preparation Strategies

The preparation of antioxidant-based MALDI matrices is a key factor determining analytical performance, as the matrix composition, concentration, homogeneity, and crystallization directly influence analyte incorporation and ionization. Conventional MALDI preparation typically involves dissolving the matrix and analyte in suitable solvents, depositing the mixture onto a target, and allowing the solvent to evaporate to induce co-crystallization. However, this procedure can lead to the formation of heterogeneous crystals and uneven analyte distribution, contributing to spot-to-spot variability [94]. These considerations are particularly relevant for natural antioxidants, as differences in solubility and intermolecular interactions can significantly affect the physical properties of the deposited matrix.

In addition to conventional manual deposition, automated matrix applicators have been developed to improve the homogeneity and reproducibility of matrix application. Automated spraying systems enable a more controlled coverage of the target surface by adjusting parameters such as the volume of solvent delivered, the spray rate, and the number of deposition cycles. Enhanced control over matrix deposition can promote more uniform crystal formation and reduce spot-to-spot variability, which is particularly important for MALDI imaging and quantitative applications [19]. Another approach is matrix deposition via sublimation, where the matrix is transferred to the target surface without the presence of a liquid solvent. Sublimation can produce a relatively homogeneous and thin matrix layer while reducing analyte delocalization associated with solvent-based deposition. Recent studies have shown that controlled thermal evaporation or sublimation can improve matrix homogeneity and contribute to more reproducible MALDI imaging performance [18]. These approaches may therefore be particularly useful for antioxidant analysis, where deposition uniformity, crystal morphology, and the preservation of analyte localization are critical analytical requirements.

The simplest approach involves using a single, purified antioxidant compound. This strategy ensures a well-defined chemical composition and facilitates the assessment of the relationship between molecular structure and matrix performance. Individual phenolic and flavonoid compounds can thus be useful for investigating the influence of specific functional groups on ionization. However, matrix performance can vary depending on solvent composition, concentration, the analyte-to-matrix ratio, and ionization polarity, necessitating the optimization of deposition conditions. Studies on flavonoid ionization have demonstrated that both matrix selection and laser intensity can significantly affect molecular ion formation and in-source fragmentation [88].

A second strategy involves binary or mixed matrices, in which two components are combined to leverage complementary properties. Such systems can be designed to enhance laser absorption, proton transfer, crystallization, or background suppression compared to single-component matrices. Similar approaches have been extensively investigated within the broader context of MALDI matrix development. For instance, graphene oxide has been combined with conventional matrices—such as DHB, CHCA, THAP, or sinapinic acid—to improve crystallization and the reproducibility of analytical results. Although these systems do not utilize matrices of antioxidant origin, they provide a useful methodological framework for evaluating combinations of natural antioxidants with additional matrix components.

A third approach employs nanostructured materials or solid particles. Nanoparticle-based systems can reduce the reliance on the conventional crystallization of organic compounds and lower background signals in the low *m*/*z* range. For instance, silicon nanoparticles have been used in LDI for small-molecule analysis, yielding significantly lower background noise in the low-mass range compared to conventional MALDI matrices [53]. Metal oxides and other nanostructured substrates have also been investigated for small-molecule analysis [54,55]. Analogous solutions can be applied to antioxidant-based systems by combining a natural antioxidant with a nanostructured substrate; however, such systems should be regarded as hybrid matrices, as their analytical properties do not stem solely from the presence of the antioxidant.

Another sample preparation strategy involves the use of antioxidant materials of natural or polymeric origin, with lignin serving as the most prominent example in this context. Zhao et al. demonstrated that lignin can function directly as an MALDI matrix for small molecules in both positive and negative ion modes—a capability confirmed, for example, through the analysis of the flavonoid myricitrin [85]. Unlike conventional small-molecule organic matrices, lignin provides a more complex and structurally heterogeneous environment that facilitates analyte incorporation and laser-induced desorption. This result indicates that useful MALDI matrices need not be limited to highly purified, small organic compounds, provided their composition and behavior during analysis have been adequately characterized.

The choice of sample preparation strategy should be tailored to the analytical objective. Single-component matrices are best suited for evaluating the impact of a specific antioxidant structure, whereas mixed or hybrid systems may prove advantageous when addressing issues related to ionization, crystallization, or background signal suppression. In MALDI-MSI, the sample preparation stage is particularly critical, as the method of matrix application can influence analyte migration and spatial resolution. Automated application and sublimation allow for better control over matrix distribution and limit solvent-induced analyte redistribution; meanwhile, nanoparticle-based strategies have been investigated for their potential to minimize conventional crystal formation and reduce analyte delocalization during matrix application [100].

Therefore, matrix preparation should be regarded as a key element in the development of antioxidant-based matrices, rather than merely a routine experimental step. Comparative studies should encompass individual antioxidant compounds as well as mixed and hybrid systems and alternative application methods, all analyzed under identical conditions; this will make it possible to determine whether observed improvements stem from the properties of the antioxidant itself or from changes made during the selection of matrix composition and application method.

### 5.4. Performance Comparison

The analytical value of an antioxidant-derived MALDI matrix cannot be established solely by the presence of detectable analyte signals. Performance should instead be assessed using parameters such as ionization efficiency, signal-to-noise ratio, background intensity, reproducibility, detection limits, and the range of compounds detected under comparable experimental conditions [8,20,84,85]. Conventional matrices provide valuable reference points for such evaluations, although reported performance values are strongly dependent on the analyte, instrument, sample preparation, and matrix-deposition procedure. For instance, CHCA has been applied to the quantitative MALDI analysis of small pharmaceutical molecules, achieving detection limits as low as 19.5 amol for the most sensitive analyte under the reported experimental conditions and enabling accurate quantification at sub-femtomole levels [101,102,103]. A separate quantitative study using DHB reports that mean relative standard deviations for several pharmaceutical analytes ranged from 6.4% to 11.1%, while lower limits of quantification varied from 4 to 100 fmol/µL [25]. These values demonstrate that conventional matrices can provide a quantitatively useful performance, but they should not be interpreted as universal matrix-specific benchmarks.

These parameters are also substantially influenced by matrix preparation. The comparative evaluation of CHCA, DHB, sinapinic acid, and other matrices demonstrated that matrix preparation affected repeatability, inter-day reproducibility, resolution, signal strength, background intensity, and analyte detectability [24]. Similarly, a systematic comparison of DHB and CHCA deposition methods for MALDI imaging showed that the application procedure had effect on both—the number of metabolites detected and analytical reproducibility [19]. Consequently, comparisons between conventional and antioxidant-derived matrices should be performed under equivalent sample-preparation, deposition, acquisition, and data-processing conditions whenever quantitative performance is reported.

For low-molecular-weight analysis, background interference may be as important as signal intensity. Conventional matrices such as CHCA and DHB can generate substantial low-*m*/*z* background, complicating the analysis of small metabolites and related compounds [5,20]. This limitation has contributed to the development of alternative matrices with different background profiles. Lignin provides a relevant example because it has been demonstrated as a matrix for small molecules in both positive and negative ion modes, showing that a high-molecular-weight phenolic material can support analyte detection while producing a spectral environment distinct from that of conventional low-molecular-weight matrices [85].

Reproducibility is another essential performance criterion. Conventional dried-droplet preparation frequently produces heterogeneous crystals, leading to variation in analyte incorporation and laser interaction across individual spots [104]. This may be particularly relevant to natural antioxidant matrices because variations in purity, solubility, molecular structure, and crystallization behavior can further increase analytical variability. Standardized preparation procedures and replicate measurements across multiple spots are therefore necessary before a candidate matrix can be considered analytically competitive.

Absolute quantification by MALDI-MS is inherently influenced by fluctuations in analyte incorporation, desorption, ionization efficiency, and matrix-related effects, which limits the reliability of raw peak intensities as direct measures of concentration. Incorporating internal standards, especially stable isotope-labelled analogues when available, can substantially enhance the reproducibility and precision of quantitative measurements but also provide means of correcting the variations in analyte recovery and ionization. Calibration may be performed using external standards, internal-standard-based calibration, or matrix-matched standards, depending on the analytical configuration and sample type. For complex biological or tissue samples, matrix-matched calibration and isotope-labeled internal standards are especially relevant because ion suppression and other matrix effects can vary across samples and tissue regions [102,103].

Even when measurements are done by the same matrix, the response factors between analytes may differ substantially, meaning that a single calibration relationship should not automatically be extrapolated across chemically different antioxidant compounds. Matrix effects and ion suppression can further alter the analytical response and also contribute to differences in sensitivity, precision, and accuracy. Therefore, intra-day and inter-day precision, calibration linearity, accuracy, recovery, and, where appropriate, dilutional performance should be evaluated using replicate measurements across relevant concentration ranges [104].

The limit of detection (LOD) and limit of quantification (LOQ) should also be determined experimentally rather than inferred from the analyte signal intensity. The quantitative study using lignin as an MALDI matrix provides an important proof of concept: six representative small molecules showed linear quantitative responses with R^2^ > 0.995, and measurements of myricitrin in Chinese bayberry samples showed strong agreement with HPLC measurements (R^2^ > 0.999) [85]. Despite that, the study should be interpreted as application-specific validation rather than evidence that lignin provides universally validated quantitative performance. For the investigated analyte and sample system, the reported result demonstrates linearity and agreement with orthogonal HPLC; however, it does not by itself establish comprehensive validation across all parameters, including intra- and inter-day precision, recovery, matrix effects, isotope-labelled internal-standard performance, and LOD/LOQ for all analytes. Hence, antioxidant-derived matrices should undergo the same quantitative validation procedures applied to conventional MALDI matrices before their analytical performance can be compared directly.

When comparing matrix systems, we should also consider fragmentation. DHB is commonly associated with relatively gentle ionization and reduced fragmentation for labile molecules, whereas sinapinic acid is frequently used when preservation of intact molecular ions from high-molecular-weight analytes is desired [15,21]. As demonstrated in experimental comparisons, matrix choice can influence fragmentation behavior; for example, alternative matrices have been reported to achieve lower detection limits together with reduced prompt fragmentation compared with DHB for acidic oligosaccharides [105]. For antioxidant-derived matrices, therefore, the detection of an analyte should be accompanied, where relevant, by an assessment of the molecular-ion preservation and product-ion formation.

Matrix stability under vacuum, storage stability, preparation time, and sample throughput are also important practical considerations. However, these parameters are inconsistently reported across studies of conventional and antioxidant-derived matrices, limiting their systematic comparison.

Where available, comparisons should also take into account the limit of quantification, dynamic range, quantitative accuracy, coefficient of variation, ion suppression, fragmentation, and matrix stability. These parameters are particularly important because a high signal intensity does not necessarily correspond to a high analytical sensitivity or quantitative reliability [24,25]. Matrix performance cannot be characterized independently of the analytical context, since ion suppression may additionally vary with analyte class and tissue sample composition [106,107]. Accordingly, performance claims for antioxidant-derived matrices should be supported by direct measurements against an appropriate conventional reference matrix rather than by absolute signal intensity alone.

Matrix selectivity and ionization polarity should also be considered. A matrix may perform effectively for acidic compounds in negative-ion mode but show poorer performance for neutral or basic analytes in positive-ion mode. Antioxidant-derived matrices should therefore be evaluated in both positive and negative ion modes when chemically appropriate. This consideration is particularly relevant to polyphenols because multiple hydroxyl and acidic functional groups can support different ionization pathways depending on the experimental conditions [88,100,108,109].

Overall, antioxidant-derived MALDI matrices should be evaluated by direct comparison with conventional reference matrices under equivalent experimental conditions. Demonstration of analyte peaks alone is insufficient; meaningful advantages should be supported by improved background suppression, ionization efficiency, reproducibility, sensitivity, or analyte compatibility. Current evidence suggests that selected naturally derived matrices may provide advantages in specific applications, but it does not support a general conclusion that they outperform established conventional MALDI matrices.

## 6. Application Areas Relevant to Antioxidant-Derived MALDI Matrices

### 6.1. Small-Molecule Metabolomics

MALDI-MS has become increasingly relevant to small-molecule metabolomics, although its application remains more challenging than the analysis of proteins and other high-molecular-weight biomolecules. One of the principal limitations is the low-*m*/*z* background generated by many conventional organic matrices, which can interfere with metabolite signals and reduce analyte coverage [17,20]. Consequently, matrix development and sample preparation strategies have increasingly focused on low-molecular-weight compounds. Rational matrix selection can substantially influence metabolome coverage because different matrices preferentially support distinct chemical classes and ionization modes [20,110].

Matrix chemistry is particularly important in negative-ion metabolomics. Comparative studies have shown that matrices such as 1,5-diaminonaphthalene can support the detection of diverse low-mass metabolites, although matrix selection also affects the relative detection of acidic metabolites, nucleotide-related compounds, and other polar species [110,111]. This is directly relevant to antioxidant analysis because antioxidant-related metabolites comprise chemically heterogeneous groups, including phenolic compounds, organic acids, and conjugated metabolites. A matrix optimized for one antioxidant class should therefore not be expected to provide equivalent performance across all antioxidant compounds.

Alternative matrix development has consequently become an important aspect of MALDI metabolomics. Approaches include modified organic matrices, high-molecular-weight materials, binary and hybrid systems, and matrices optimized for negative- or dual-polarity analysis [20,112]. More recently, rational and computational strategies have been investigated to identify structures with favorable electron-transfer properties for small-molecule MALDI [86]. Such approaches are relevant to antioxidant-derived matrices because many antioxidants contain conjugated and redox-active systems that may influence electron- or proton-transfer processes. Nevertheless, antioxidant activity alone cannot be used to predict MALDI performance. Chemical derivatization represents another important strategy for enhancing the MALDI-MS analysis of small-molecule compounds—particularly metabolites characterized by a low ionization efficiency or low abundance. Derivatization reagents enable the introduction of functional groups that facilitate protonation or deprotonation, increase ionization efficiency, or improve the detectability of specific classes of metabolites. In MALDI-MSI, the feasibility of performing chemical derivatization directly on tissue sections has also been investigated to improve the detection and spatial visualization of metabolites that are otherwise difficult to detect directly [112]. Such approaches may be particularly significant for metabolites with antioxidant properties, including chemically diverse phenolic compounds, organic acids, and other substances with varying ionization efficiencies. However, it should be noted that derivatization entails an additional sample preparation step and can affect analyte recovery, reaction selectivity, spatial localization, and the interpretation of quantitative results. Therefore, derivatization should be viewed as a complement to matrix optimization rather than a substitute for the proper selection of the matrix itself.

MALDI-MS has also been applied to complex metabolite mixtures and natural-product extracts, where rapid molecular profiling provides a useful complement to chromatographic methods. This is particularly relevant to antioxidant-rich materials, which commonly contain multiple structurally related compounds. MALDI-based profiling can therefore support sample discrimination and exploratory characterization, although structural confirmation and quantitative analysis often require complementary LC-MS or MS/MS methods [17,113].

The integration of MALDI with spatial metabolomics further expands its analytical value. MALDI-MSI enables metabolites to be detected directly within tissue sections while preserving spatial information that is lost during conventional extraction [114]. MALDI-2 post-ionization has further improved the detection of low-molecular-weight metabolites, including compounds below *m*/*z* 600, by increasing the post-ionization efficiency [115]. These developments are particularly relevant to antioxidant studies in which tissue localization may be as important as overall abundance.

Nevertheless, quantitative interpretation remains a challenge. The signal intensity in MALDI is influenced by the crystallization process, the mode of analyte incorporation into the matrix, ion suppression, compound-specific ionization characteristics, and spot-to-spot variability; consequently, the signal cannot be treated as a direct measure of metabolite concentration [17,20,116].

Therefore, for quantitative applications, analytical parameters—such as repeatability, sensitivity, limits of detection and quantification (LOD and LOQ), linearity, and accuracy—should be evaluated. The reliability of quantitative results can be enhanced by developing methods for more uniform matrix deposition, using appropriate internal standards, optimizing sample preparation, and controlling data acquisition conditions. Even so, MALDI-MS is generally best suited for exploratory studies (profiling) and spatial metabolomics, whereas the quantitative analysis of antioxidants may benefit from the use of internal standards and complementary chromatographic methods.

Overall, small-molecule metabolomics represents a significant application area for antioxidant-derived matrices within the broader development of MALDI-based metabolomics. Advances in matrix design, chemical derivatization, controlled matrix deposition, reproducibility, and quantitative analysis have expanded the analytical capabilities of MALDI regarding small molecules. Consequently, the analytical value of antioxidant-derived matrices depends not only on minimizing low-*m*/*z* interference and ensuring efficient ionization but also on achieving reproducible results, adequate sensitivity, and compatibility with quantitative and spatial analysis procedures in metabolomics. These requirements provide a crucial framework for evaluating natural antioxidant compounds as potential candidates for MALDI matrices.

### 6.2. Lipidomics

MALDI-MS is a well-established tool in lipidomics because many lipid classes can be detected with limited fragmentation and the technique enables the rapid analysis of complex lipid mixtures and tissue sections [5,117]. This is relevant to antioxidant research because oxidative processes involve polyunsaturated fatty acids, phospholipids, and lipid oxidation products.

Matrix selection is a major determinant of lipid detection. Different lipid classes may exhibit substantially different ionization efficiencies depending on the selected matrix, and inappropriate matrix conditions can lead to the poor detection of specific lipid species [5,117]. Matrix choice therefore influences both sensitivity and the range of lipid classes detected [5]. This is particularly relevant to oxidized or antioxidant-associated lipids, whose polarity and functional groups may alter their interactions with the matrix. MALDI-MSI further enables the spatial visualization of lipid species and may support investigations of antioxidant-associated lipids or lipid oxidation products [118,119,120]. Quantitative lipidomics using MALDI nevertheless remains challenging. Signal intensities are affected by ion suppression, variable ionization efficiencies, adduct formation, and matrix-dependent effects, which complicate the direct quantitative comparison among lipid species [5,118]. Therefore, accurate quantification requires appropriate internal standards or complementary LC-MS approaches.

Alternative matrix development remains relevant to lipidomics. Current approaches include modified organic matrices, uniform matrix layers, and nanostructured materials designed to improve reproducibility and reduce matrix-derived interference [5,121]. Although most established lipid matrices are not antioxidant-derived, these approaches provide a framework for evaluating natural antioxidant candidates.

### 6.3. Natural Product Profiling

Natural products represent an important application area for MALDI-MS because plant extracts and other biological materials contain chemically diverse mixtures of secondary metabolites that can be difficult to characterize using a single analytical technique [122,123,124,125,126]. MALDI-TOF MS has been used for the rapid profiling of alkaloids, flavonoids, carotenoids, tannins, and other small molecules with limited sample preparation [60,127,128]. This enables the generation of molecular fingerprints that can be compared across species, tissues, extracts, and processing conditions.

MALDI has also been applied specifically to antioxidant-rich plant compounds. Carotenoids have been detected directly from plant cells and crude extracts using MALDI-TOF MS, enabling a rapid profiling and semi-quantitative assessment [129]. MALDI-MSI has also been used in plant tissues containing flavonoids, tannins, phenolic compounds, and other secondary metabolites, allowing their spatial distribution to be examined directly [130,131]. This spatial information is particularly relevant when biological function depends on tissue localization rather than total abundance alone.

A further advantage of MALDI in natural-product research is the combination of molecular profiling with spatial analysis. MALDI-MSI has been used to map a broad range of secondary metabolites in medicinal plants, including alkaloids, coumarins, sugars, organic acids, flavonoids, and lipids [130,131,132]. In *Paeonia* roots, high-resolution MALDI-MSI enabled the spatial characterization of flavonoids, tannins, glycosides, and other metabolites, while tandem MSI supported the differentiation of structurally related compounds [130]. Such approaches provide localization information that cannot be obtained from homogenized extracts.

When a greater chemical specificity is required, MALDI can be combined with chromatographic separation. TLC-MALDI-TOF MS has been applied to the characterization and dereplication of flavonoids in plant-derived samples including wine, apple, and rose [68]. This approach combines chromatographic resolution with mass-based molecular information. MALDI-TOF MS/MS has also been used for the rapid screening and quantitative analysis of plant compounds, further supporting its role as a complement rather than a replacement for LC-MS [133].

Matrix choice is especially important for chemically complex natural-product samples. Composite matrix systems have been developed to improve the MALDI-MSI of plant secondary metabolites. For example, submicron 3,4-dihydroxybenzoic acid–TiO_2_ composite particles have been used for imaging secondary metabolites in *Scutellaria baicalensis* roots [134]. Such systems demonstrate how phenolic structures can be incorporated into alternative matrix formulations, although they do not establish antioxidant activity itself as the determinant of matrix performance.

Overall, MALDI-MS provides a useful platform for rapid natural-product fingerprinting, antioxidant profiling, and the spatial mapping of plant metabolites. Antioxidant-derived matrices may be particularly relevant in this area if they improve the detection of phenolic compounds and other low-molecular-weight metabolites without compromising reproducibility or spectral clarity.

### 6.4. Food Authenticity and Quality Control

MALDI-MS has attracted interest in food analysis because it enables the rapid molecular profiling of complex samples with relatively limited preparation. This is particularly relevant to antioxidant-rich foods containing diverse phenolic compounds, flavonoids, carotenoids, and other metabolites that contribute to nutritional and sensory properties. Rather than focusing on a single compound, MALDI-based profiling can generate broader molecular fingerprints useful for comparing products, varieties, origins, and processing conditions [127,135,136,137,138,139,140].

One important application is food authentication and discrimination. MALDI-TOF MS has been used to profile low-molecular-weight compounds in plant-derived foods and beverages, producing chemical fingerprints suitable for multivariate analysis. In wine, for example, mass-spectral profiling has been investigated for differentiation according to composition and origin [141]. Similar approaches have been applied to plant materials and other food products, demonstrating that MALDI profiles can contain sufficient molecular information to distinguish chemically different samples [127,142]. This is particularly relevant to antioxidant-rich foods, where phenolic and metabolite profiles vary with cultivar, geographical origin, and processing.

MALDI-MS has also been used for phenolic and flavonoid analysis in food matrices. Direct TLC-MALDI-TOF MS has enabled the characterization of flavonoids in complex plant-derived samples, including wine and fruit extracts [68]. This workflow combines chromatographic separation with mass-based identification and can reduce ambiguity in direct mixture analysis. MALDI-MSI provides an additional option for solid foods and plant tissues by enabling the spatial visualization of metabolites rather than the measurement of average concentrations after extraction [131].

Quality control during processing and storage represents another potential application. Food processing can alter antioxidant composition and other metabolic profiles, and mass-spectrometric fingerprinting may detect changes associated with heating, fermentation, drying, and other processing conditions [140,142]. MALDI profiling can therefore serve as a rapid screening approach based on overall molecular patterns rather than individual predefined markers.

Food analysis nevertheless presents substantial analytical challenges. Salts, sugars, lipids, pigments, and other matrix components can influence extraction and ionization [127,140]. Matrix effects may reduce reproducibility and complicate quantitative comparison, while structurally related phenolic compounds may be difficult to distinguish using accurate mass alone. MALDI fingerprints should therefore be interpreted together with MS/MS, chromatographic separation, or other orthogonal methods when definitive compound identification or quantification is required.

These limitations provide a rationale for investigating antioxidant-derived MALDI matrices in food analysis. Naturally occurring phenolic compounds may offer useful matrix-related chemical properties, but their antioxidant origin does not guarantee improved analytical performance. Candidate matrices should therefore be assessed through direct comparisons of sensitivity, background interference, reproducibility, and analyte coverage.

Overall, MALDI-MS is particularly suited to the rapid screening, molecular fingerprinting, authentication, classification, and monitoring of antioxidant-rich food products. Alternative matrix development may further expand its utility for the targeted analysis of phenolic antioxidants and other low-molecular-weight food constituents.

## 7. Mechanistic Insights

The performance of an MALDI matrix results from the interaction of its chemical and physical properties with laser radiation and the analyte during desorption and ionization. Although several mechanisms have been proposed, the relative contribution of individual processes remains dependent on the matrix, analyte, and experimental conditions, and MALDI ion formation cannot be explained by a single universal mechanism [9,10]. The potential of a natural antioxidant to function as an MALDI matrix therefore cannot be inferred from antioxidant activity or any single physicochemical property alone. Instead, its optical, acid–base, redox, and physical properties should be regarded as potentially contributing factors that require experimental validation in the relevant matrix–analyte system.

**Laser Energy Absorption**

Efficient absorption at the laser wavelength is a fundamental requirement for conventional MALDI matrices. Organic compounds containing aromatic and conjugated structures can absorb UV radiation and may contribute to energy deposition relevant to desorption and ionization [9,15]. Many natural antioxidants, particularly phenolic acids and flavonoids, contain conjugated aromatic systems and therefore possess optical properties that make them plausible candidates for investigation as laser-absorbing matrix materials. However, efficient UV absorption alone does not guarantee effective matrix performance, because successful ionization also depends on the broader matrix–analyte interaction and experimental conditions [10].

**Proton and Electron Transfer**

Matrix–analyte interactions may involve proton transfer, deprotonation, and electron-transfer reactions. These processes may be particularly relevant to low-molecular-weight compounds, whose ionization efficiency can vary substantially according to chemical structure [10,13]. Phenolic hydroxyl, carboxyl, and other ionizable groups present in many natural antioxidants may therefore influence the formation of protonated or deprotonated analyte ions. Computational approaches have also indicated that electron-transfer properties can be incorporated into rational MALDI matrix design, further emphasizing the importance of the electronic structure in matrix selection [86].

**Redox Properties**

Although antioxidants are inherently associated with redox chemistry, oxidation or reduction potential should not be interpreted as a direct indicator of superior MALDI matrix performance. Redox properties may support particular ionization pathways, but their contribution depends on the analyte and experimental conditions [17,86]. This distinction is especially relevant to polyphenols, whose antioxidant activity reflects their chemical reactivity toward radical species, whereas MALDI ionization involves a broader combination of photophysical, acid–base, and charge-transfer processes.

**Matrix–Analyte Interactions and Crystal Morphology**

The physical organization of the matrix is also an important determinant of MALDI performance. During dried-droplet preparation, matrix–analyte co-crystallization influences analyte distribution and the consistency of laser interaction across the sample spot [109]. Crystal morphology can therefore affect signal intensity and reproducibility independently of matrix chemistry. Natural antioxidants may differ considerably in solubility and crystallization behavior, making the optimization of solvent composition, concentration, matrix-to-analyte ratio, and deposition conditions necessary when evaluating them as potential matrices.

Overall, the performance of antioxidant-derived matrix candidates is likely to depend on several interconnected properties, including laser absorption, proton- and electron-transfer behavior, redox characteristics, and matrix–analyte organization. Therefore, these properties should be regarded as contributing factors and not as independent predictors of matrix performance. Specifically, antioxidant capacity, reduction potential, proton-donating ability, aromaticity, or UV absorption alone cannot establish matrix suitability in the absence of experimental evidence. Further mechanistic studies are therefore needed to determine which structural and physicochemical characteristics consistently support efficient and reproducible MALDI ionization under defined analytical conditions.

An additional strategy for enhancing MALDI ionization is laser-induced post-ionization (MALDI-2), in which a second laser pulse is applied to the desorption plume to promote additional gas-phase ionization. MALDI-2 can increase ion yields and improve the detection of low-molecular-weight metabolites and other small molecules compared with conventional MALDI [115,139]. This approach represents an ionization-enhancement strategy rather than a matrix class itself and may therefore complement the use of alternative matrix materials such as antioxidant-derived candidates.

## 8. Comparison with Emerging Matrix Technologies

The limitations of conventional organic matrices in low-molecular-weight MALDI analysis have encouraged the development of alternative matrix technologies, including modified organic matrices, ionic-liquid systems, polymers, nanomaterials, carbon-based materials, and matrix-free surfaces [20]. These approaches are primarily intended to reduce low-*m*/*z* background, improve ionization, and enhance analytical reproducibility. Antioxidant-derived matrices should be assessed within this broader context and compared directly with established and emerging matrix technologies rather than evaluated solely on the basis of antioxidant activity. The main characteristics of these approaches and their relevance to antioxidant-derived MALDI matrices are summarized in Table 3.

## 9. Challenges and Current Limitations

Although MALDI-MS has become increasingly important in small-molecule analysis, several limitations still restrict its broader analytical and quantitative application. These challenges are particularly relevant when natural antioxidants are considered as alternative matrices because their chemical diversity and physicochemical properties may introduce additional variability. Conventional matrices can generate interfering signals in the low-*m*/*z* region, while analyte ionization remains strongly dependent on matrix composition, sample preparation, and crystallization [1,2]. Therefore, antioxidant-derived matrix candidates should be evaluated not only on the basis of detectable analyte signals but also in terms of reproducibility, selectivity, quantitative performance, and compatibility with complex samples. The main challenges associated with their wider application are summarized in Table 4.

## 10. Future Perspectives

The future development of MALDI-MS for antioxidant analysis will likely depend on improving matrix performance while making matrix selection more systematic and reproducible. Recent studies have increasingly shifted from empirical screening toward rational and computationally guided matrix design, including approaches that consider electronic and structural properties relevant to low-molecular-weight ionization [20,86]. This direction may be particularly useful for natural antioxidants because their structural diversity provides a broad chemical space for identifying potential matrix candidates.

Standardization and high-throughput workflows will also be important. The improved control of matrix deposition, crystallization, and instrumental conditions is needed to enhance reproducibility and enable the meaningful comparison of matrix performance across laboratories [116,148]. Automated sample preparation and imaging platforms may further increase the applicability of MALDI to large sets of biological, food, and natural-product samples.

The integration of MALDI-MS with metabolomics and mass spectrometry imaging is also expected to remain important. Developments such as MALDI-2 have improved the detection of low-molecular-weight metabolites and lipids, while high-resolution MSI increasingly enables molecular distributions to be examined in relation to tissue morphology [76,139]. In antioxidant research, these approaches may support not only compound identification but also investigation of spatial distribution and metabolic relationships.

Natural antioxidant-derived matrices represent a particularly relevant direction within this broader development. Future studies should focus on establishing structure–property–performance relationships rather than assuming that antioxidant activity itself predicts MALDI suitability. Features such as aromaticity, conjugation, acidity, redox potential, UV absorption, solubility, and crystallization behavior should be evaluated systematically in relation to ionization performance. Computational approaches may help prioritize compounds for experimental testing and reduce the need for purely empirical screening [20,86].

Broader analytical use will ultimately require stronger validation, particularly for quantitative applications. A direct comparison with established MALDI matrices and LC-MS methods, together with appropriate internal standards and standardized performance metrics, will be necessary before antioxidant-derived matrices can be considered reliable for routine analysis [148,149]. Future progress is therefore more likely to involve a set of matrix systems optimized for specific antioxidant classes, sample types, and analytical objectives than a single universally applicable antioxidant-derived matrix.

## 11. Conclusions

MALDI-MS is a versatile analytical platform for small molecules, natural products, lipids, pharmaceuticals, and biological samples. Its application to low-molecular-weight compounds, however, remains limited by matrix-derived background, ion suppression, variable ionization efficiency, and reproducibility. These limitations have driven the development of alternative matrix technologies and have highlighted the central role of matrix chemistry in determining analytical performance.

Natural antioxidants represent an emerging and still insufficiently explored group of potential MALDI matrix candidates. Their aromatic and conjugated structures, together with ionizable functional groups, may provide properties relevant to laser absorption, matrix–analyte interactions, and ionization. However, antioxidant activity alone does not determine matrix suitability. Candidate matrices must be evaluated according to background generation, ionization efficiency, reproducibility, analyte coverage, crystallization behavior, and quantitative performance.

The available evidence also indicates that no single matrix is likely to be optimal for all antioxidant compounds or sample types. Future progress will therefore depend on systematic matrix selection, standardized preparation procedures, mechanistic investigation, and rational or computational matrix design. Integration with metabolomics, MS/MS, and MALDI-MSI may further expand the analytical information obtainable from antioxidant-rich biological, plant, and food samples.

Overall, antioxidant-derived matrices should be regarded as a complementary strategy within the broader development of MALDI-MS rather than as an immediate replacement for established conventional matrices or LC-MS-based workflows. Establishing clear structure–performance relationships and validating promising candidates against established analytical methods will be essential for determining their practical value in antioxidant analysis and other low-molecular-weight applications.

## Figures and Tables

**Table 2:** Experimentally demonstrated antioxidant-related and naturally derived materials used as MALDI matrices.

Material	Chemical Class	Matrix Status	Demonstrated Application	Reference
Sinapinic acid	Hydroxycinnamic acid	Established conventional matrix	Proteins, high-MW analytes	[4,12,15]
2,5-Dihydroxybenzoic acid	Dihydroxybenzoic acid	Established conventional matrix	Oligonucleotides	[96]
Ferulic acid	Hydroxycinnamic acid	Established matrix	oligonucleotides	[96]
Caffeic acid	Hydroxycinnamic acid	Experimentally demonstrated antioxidant-derived matrix	HMW proteins, MALDI-MSI	[97]
Curcumin	Curcuminoid/polyphenol	Experimentally demonstrated antioxidant-derived matrix	drugs, lipids, MALDI-MSI	[98]
Lignin	Phenolic polymer	Experimentally demonstrated naturally derived matrix	Small molecules; quantitative analysis	[85]
Quercetin	Flavonoid	Experimentally demonstrated antioxidant-derived matrix	Endogenous lipids, negative-ion MALDI-MSI	[99]

**Table 3:** Comparison of conventional, emerging, and antioxidant-derived MALDI matrix technologies.

Matrix Technology	Main Principle/Characteristics	Advantages	Main Limitations	Relevance to Antioxidant-Derived Matrices
Conventional organic matrices	Small UV-absorbing organic molecules such as CHCA and DHB facilitate analyte desorption and ionization.	Well established; efficient for many biomolecules; widely available.	Strong background in the low-*m*/*z* region; matrix–analyte interference.	Provide the principal benchmark for evaluating alternative antioxidant-derived matrices [17,20].
Rationally modified organic matrices	Chemical modification of established matrices to alter volatility, ionization behavior, or spectral background.	Can retain favorable optical properties while improving performance in the low-*m*/*z* region.	Requires structural optimization; performance remains analyte-dependent.	Provide a useful framework for modifying antioxidant structures while retaining relevant functional groups [20,112].
Ionic-liquid matrices	Ionic derivatives of conventional matrices provide more homogeneous analyte incorporation and reduce conventional crystallization.	Reduced crystallization heterogeneity; potentially improved reproducibility and ionization.	Preparation can be more complex; performance remains matrix-specific.	Natural antioxidant compounds could potentially be incorporated into ionic matrix systems [143].
Polymeric/high-molecular-weight matrices	Larger conjugated structures provide laser absorption while generating fewer low-*m*/*z* matrix-derived ions.	Reduced low-*m*/*z* background; good vacuum stability.	More complex preparation; not universally suitable for all analytes.	Relevant to naturally derived high-molecular-weight phenolic materials and background-suppression strategies [112].
Nanomaterial-assisted matrices	Metals, semiconductors, graphene, carbon nanotubes, or related materials facilitate laser energy transfer and/or analyte desorption.	Low background; tunable physicochemical properties; useful for small molecules.	Surface preparation and reproducibility can be challenging; mechanisms vary among materials.	Antioxidants may be combined with nanomaterials to form hybrid matrix systems [144,145].
Graphene/carbon-based materials	Carbon nanostructures absorb laser energy and support desorption and ionization.	Broad chemical compatibility; relatively low background compared with conventional matrices.	Surface functionalization and analyte adsorption may affect reproducibility.	May provide complementary properties in hybrid antioxidant–carbon systems [145,146].
Matrix-free LDI	Structured silicon or nanostructured surfaces directly promote desorption/ionization without a conventional organic matrix.	Minimal matrix-derived background; particularly attractive for low-*m*/*z* compounds.	Requires specialized substrates; analyte compatibility can be limited.	Represents an alternative strategy rather than a direct antioxidant-derived matrix approach [147].
Antioxidant-derived matrices	Selected natural antioxidant compounds are investigated as potential UV-absorbing and chemically active MALDI matrices.	May combine aromatic or conjugated structures with functional groups relevant to laser absorption and ionization.	Experimental evidence remains limited; reproducibility, purity, crystallization, background generation, and matrix–analyte interactions require systematic validation.	Central focus of this review and an emerging strategy requiring direct comparison with established matrix systems [20,112].

**Table 4:** Current challenges and limitations associated with natural antioxidant-derived MALDI matrices.

Challenge	Analytical Significance	Specific Concern for Antioxidant-Derived Matrices	Potential Approach
Low-*m*/*z* background interference	Matrix-derived ions may overlap with low-molecular-weight analytes and reduce detectable metabolite coverage.	Some antioxidant compounds may themselves generate matrix-derived ions or clusters within the analytical mass range.	Development of larger, modified, or low-background antioxidant-derived matrix systems [17,20].
Reproducibility	Differences in crystallization and analyte distribution can cause spot-to-spot and sample-to-sample variability.	Natural compounds may show variable crystallization depending on purity, solvent, concentration, and deposition conditions.	Standardized preparation, deposition, and crystallization protocols [109,116].
Purity and batch variability	Impurities can introduce additional signals and alter matrix performance.	Naturally sourced antioxidant materials may vary with botanical origin, cultivation, extraction, and processing.	Use of well-characterized purified compounds and clearly defined material specifications [109,116].
Matrix–analyte selectivity	A matrix optimized for one chemical class may perform poorly for another.	Antioxidants include structurally diverse phenolic acids, flavonoids, carotenoids, and related compounds.	Evaluation against chemically diverse analyte panels rather than individual model compounds [17,20].
Quantitative analysis	Ionization efficiency and matrix effects may prevent direct correlation between signal intensity and analyte concentration.	Different antioxidants may exhibit substantially different response factors within the same matrix system.	Internal standards, appropriate calibration strategies, and MS/MS-based quantification [148,149].
Sample preparation	Extraction conditions, solvent composition, concentration, and deposition influence analyte recovery and ionization.	Complex natural extracts may contain salts, sugars, lipids, and other components that alter matrix performance.	Optimized extraction procedures and controlled matrix deposition [17,20].
Mechanistic uncertainty	The relative contribution of proton transfer, electron transfer, energy absorption, and desorption depends on the matrix–analyte system.	Antioxidant activity alone does not predict MALDI matrix performance.	Structure–performance studies and computationally guided matrix design [17,20].
Standardization and validation	Lack of standardized protocols limits comparison across laboratories and instruments.	Different antioxidant-derived matrices may require substantially different preparation and acquisition conditions.	Interlaboratory validation and standardized analytical workflows [116,148].
Complex biological and food matrices	Ion suppression and chemical competition may reduce sensitivity and complicate compound identification.	Antioxidant-rich samples contain numerous structurally related compounds and other potential interferents.	Separation, derivatization, MS/MS, or complementary LC-MS analysis [17,150].

## Data Availability

No new data were created or analyzed in this study. Data sharing is not applicable to this article.

## Abbreviations

The following abbreviations are used in this manuscript:

AI	Artificial intelligence
CHCA	α-Cyano-4-hydroxycinnamic acid
DHB	2,5-Dihydroxybenzoic acid
GC-MS	Gas chromatography–mass spectrometry
HPLC	High-performance liquid chromatography
HPLC-ESI-MS	High-performance liquid chromatography–electrospray ionization mass spectrometry
HPTLC	High-performance thin-layer chromatography
LC-MS	Liquid chromatography–mass spectrometry
LDI	Laser desorption/ionization
LOD	Limit of detection
LOQ	Limit of quantification
MALDI	Matrix-Assisted Laser Desorption/Ionization; an ionization technique used to generate ions from matrix–analyte samples
MALDI-2	Matrix-assisted laser desorption/ionization with laser post-ionization
MALDI-MSI	Matrix-Assisted Laser Desorption/Ionization Mass Spectrometry Imaging; an application of MALDI-MS that provides spatially resolved molecular information from a sample.
MALDI-MS	Matrix-Assisted Laser Desorption/Ionization Mass Spectrometry; mass spectrometric analysis using MALDI as the ionization technique, without specifying the type of mass analyzer.
MALDI-TOF	Matrix-assisted laser desorption/ionization time-of-flight
MALDI-TOF-MS	Matrix-Assisted Laser Desorption/Ionization Time-of-Flight Mass Spectrometry; a mass spectrometric configuration combining MALDI ionization with a TOF mass analyzer.
MS	Mass Spectrometry; an analytical technique comprising an ionization source, a mass analyzer, and a detector.
MS/MS	Tandem mass spectrometry
MSI	Mass spectrometry imaging
MOF	Metal–organic framework
Nd:YAG	Neodymium-doped yttrium aluminum garnet
NMR	Nuclear magnetic resonance
RSD	Relative standard deviation
SA	Sinapinic acid
THAP	2′,4′,6′-Trihydroxyacetophenone
TLC	Thin-layer chromatography
TLC-MALDI-TOF	Thin-layer chromatography–matrix-assisted laser desorption/ionization time-of-flight
TOF	Time-of-Flight; a type of mass analyzer that separates ions according to their flight times.
UV	Ultraviolet
